# Transcriptome-wide mRNP condensation precedes stress granule formation and excludes new mRNAs

**DOI:** 10.1016/j.molcel.2025.11.003

**Published:** 2025-11-24

**Authors:** Hendrik Glauninger, Jared A.M. Bard, Caitlin J. Wong Hickernell, Karen M. Velez, Edo M. Airoldi, Weihan Li, Robert H. Singer, Sneha Paul, Jingyi Fei, Tobin R. Sosnick, Edward W.J. Wallace, D. Allan Drummond

**Affiliations:** 1 Graduate Program in Biophysical Sciences, The University of Chicago, Chicago, IL, USA; 2 Interdisciplinary Scientist Training Program, The University of Chicago, Chicago, IL, USA; 3 Department of Biology, Texas A&M University, College Station, TX, USA; 4 Department of Biochemistry & Molecular Biology, The University of Chicago, Chicago, IL, USA; 5 Department of Molecular Genetics & Cell Biology, The University of Chicago, Chicago, IL, USA; 6 Fox School of Business and Management, Temple University, Philadelphia, PA, USA; 7 Department of Cell Biology, Albert Einstein College of Medicine, Bronx, New York, NY, USA; 8 Gruss-Lipper Biophotonics Center, Albert Einstein College of Medicine, Bronx, New York, NY, USA; 9 Department of Neuroscience, Albert Einstein College of Medicine, Bronx, New York, NY, USA; 10 Institute for Biophysical Dynamics, The University of Chicago, Chicago, IL, USA; 11 Pritzker School of Molecular Engineering, The University of Chicago, Chicago, IL, USA; 12 School of Biological Sciences, University of Edinburgh, Edinburgh, Scotland, UK; 13 Department of Medicine, Section of Genetic Medicine, The University of Chicago, Chicago, IL, USA; 14 Present address: Department of Biology, North Park University, Chicago, IL, USA; 15 These authors contributed equally; 16 Senior author; 17 Lead contact

## Abstract

Stress-induced messenger ribonucleoprotein (mRNP) condensation is conserved across eukaryotes, resulting in stress granule formation under intense stresses, yet the mRNA composition and function of these condensates remain unclear. Exposure of ribosome-free mRNA following stress is thought to cause condensation and stress granule formation through mRNA-sequence-dependent interactions, leading to disproportionate condensation of long mRNAs. Here, we show that, by contrast, virtually all mRNAs condense in response to multiple stresses in budding yeast with minor length dependence and often without stress granule formation. New transcripts escape mRNP condensation, enabling their selective translation. Inhibiting translation initiation causes formation of mRNP condensates distinct from stress granules and processing bodies (P bodies), and these translation-initiation-inhibited condensates (TIICs) are omnipresent, even in unstressed cells. Stress-induced mRNAs are excluded from TIICs due to the timing of their expression, indicating determinants of escape that are independent of sequence. Together, our results reveal a previously undetected level of translation-linked molecular organization and stress-responsive regulation.

## In brief

Glauninger, Bard, Wong Hickernell, et al. reveal that stress causes transcriptome-wide mRNP condensation, largely independent of length and often without stress granule formation, but newly synthesized mRNAs escape this sequestration, enabling their selective translation. Translation initiation inhibition causes condensation of mRNA, and these condensates are present even in unstressed cells.

## Graphical Abstract

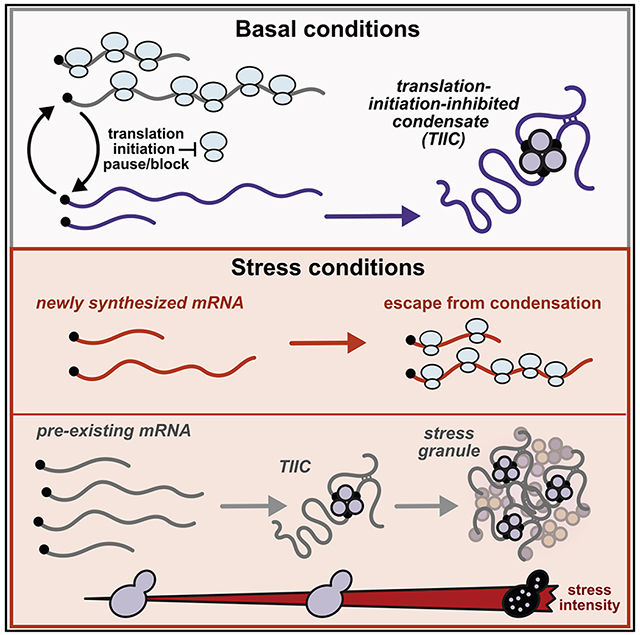

## INTRODUCTION

Cells must respond to changing environments to survive and thrive. When faced with a broad range of sudden maladaptive environmental changes—stresses—eukaryotic cells downregulate translation, induce stress-responsive transcriptional programs, and form cytosolic clusters of mRNA and proteins. When microscopically visible as foci colocalized with markers such as poly(A)-binding protein, these clusters are called stress granules (SGs).^1–7^ Conserved across eukarya, SGs are complex examples of biomolecular condensates, membraneless structures without defined stoichiometry that form by a range of processes and that concentrate specific types of biomolecules.^8–10^ Their function remains unclear, as does the relationship between SG formation and the accompanying transcriptional and translational responses.^11^

Early work in multiple systems established that what are now recognized as SGs recruit multiple RNA-binding proteins along with pre-stress mRNA, yet exclude mRNA produced during stress.^12,13^ In mammalian cells, SGs were shown to nonspecifically recruit untranslated mRNA but exclude two specific stress-induced mRNAs, HSP70 and HSP90.^14,15^ This matched prior work on heat-shocked granules in plants, which recruited mRNA encoding housekeeping proteins but not mRNA encoding newly synthesized heat shock proteins.^6^ In glucose-starved yeast cells, induced transcripts include those that are translationally repressed and accumulate in foci and others that are soluble and translated.^16^ More recent work finds most translationally repressed mRNA outside visible foci.^17^

Stress-triggered inhibition of translation initiation plays a central role in SG formation.^13^ Subsequent ribosome runoff, polysome disassembly, and the exposure of ribosome-free mRNA have been proposed to serve as a template or “universal trigger” for SG assembly.^13,18–20^ Consistent with this model, inhibitors of translation elongation that lock ribosomes on transcripts, such as cycloheximide (CHX) and emetine, inhibit SG formation, whereas an elongation inhibitor that causes ribosome release, puromycin, promotes SG formation.^18,21^

Measurements of the mRNA enriched in SGs have been interpreted as supporting a central role of ribosome-free RNA in SG formation, as in both yeast and mammalian cells mRNA length appeared to be the dominant predictor of SG targeting.^4,22–24^ Furthermore, increasing RNA length promotes RNA/protein phase separation *in vitro* by the stress granule hub protein G3BP1,^25,26^ and single-molecule studies show that mRNA length correlates with dwell time on SGs and other condensed structures.^27^ These results fit a model in which long RNAs provide more opportunities for multivalent interactions necessary to form condensates. Yet these transcriptome-scale findings are in apparent conflict with results showing selective exclusion of stress-induced mRNA from SGs.^11^

By contrast, other work highlights a central role for protein components in mRNA-protein condensation and SG formation.^5,26,28–33^ In mammalian cells, the protein-protein interaction network mediated by G3BP1/2 is critical for SG assembly following arsenite treatment.^29^ Meanwhile, blocking visible SG formation with CHX does not block *in vivo* condensation of Pab1,^34^ and mild stresses trigger protein condensation without SG formation.^34,35^ Indeed, multiple RNA-binding proteins, including SG markers, autonomously condense *in vivo* and *in vitro* in response to physiological stress conditions.^7,34,36–38^ This supports a model in which stages of protein condensation occur regardless of whether visible SGs eventually appear.^11^ Whether a staged model applies to mRNA condensation remains to be studied.

Here, using a wide range of methods including biochemical fractionation by sedimentation and RNA sequencing (Sed-seq), we show that virtually all pre-stress transcripts condense during stress regardless of their lengths, even in the absence of visible SGs. At the transcriptome scale, stress-induced transcripts escape condensates and are robustly translated. A simple explanation rationalizes stress-specific differences in condensed mRNA: preexisting transcripts condense, and newly produced transcripts escape condensation, permitting their preferential translation. We discover that specific endogenous transcripts are condensed prior to stress, only to be released from condensates to be translated during stress. Most surprisingly, condensation is pervasive even in unstressed cells and results from inefficient translation initiation. These translationinitiation-inhibited condensates (TIICs) contain both mRNA and protein, are distinct from SGs, and potentiate SG formation. Together, these results show that mRNA-protein condensation occurs even basally outside of stress and is measurable before visible SGs form, expanding the importance of understanding mRNA-protein condensation for cellular physiology in and outside of stress.

## RESULTS

### Sed-seq enables measurement of transcriptome-scale mRNA condensation

**Figure 1.**
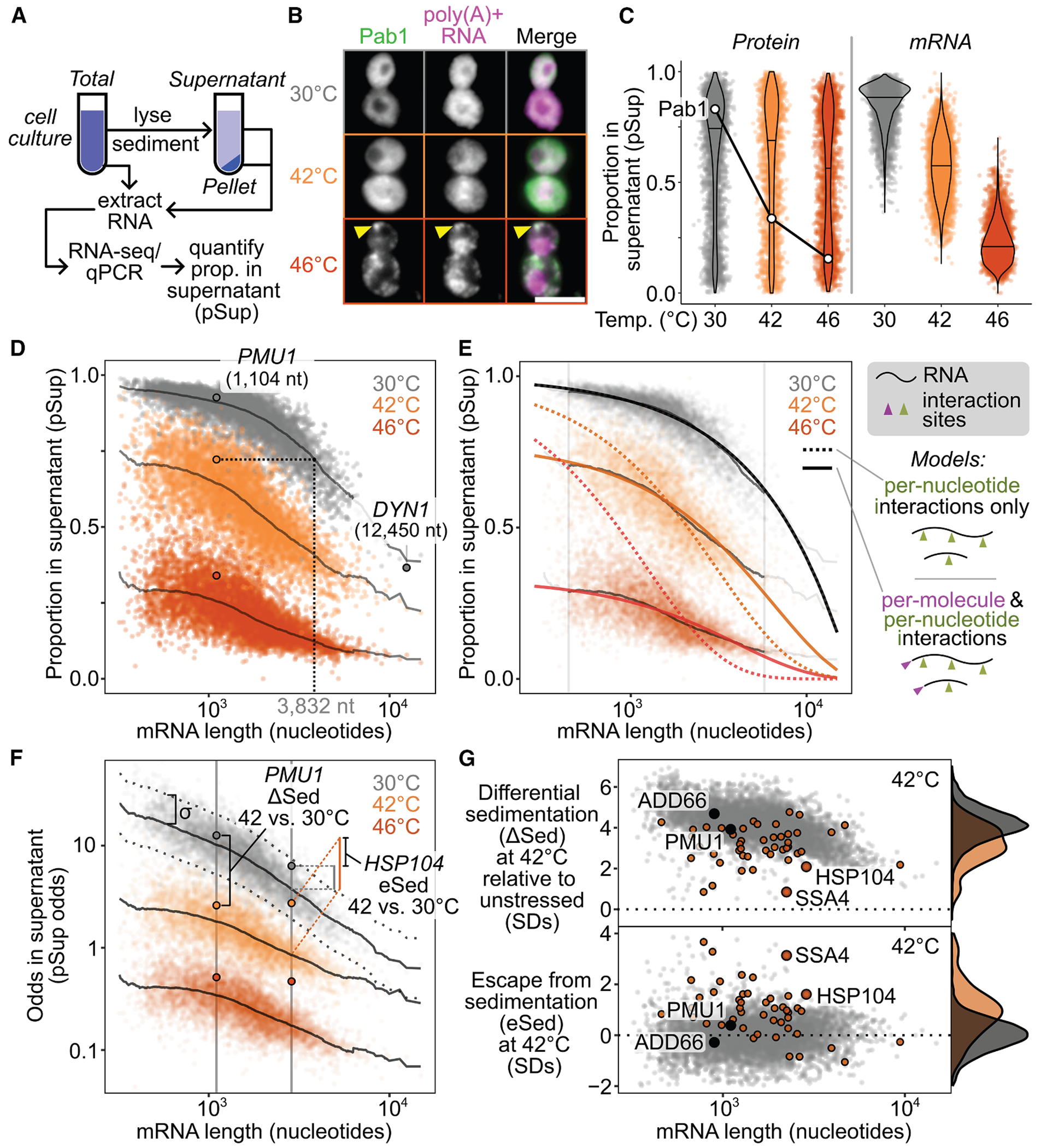
Most transcripts condense during stress, even in the absence of SGs (A) Analysis of mRNA condensation by sedimentation and RNA sequencing (Sed-seq) enables calculation of mRNA proportion in the supernatant (pSup) across conditions. (B) 15 min of heat shock induces SG formation at 46°C but not at 42°C, as marked by poly(A)-binding protein (Pab1-HaloTag) and FISH against poly(A)+ RNA (scale bar, 5 μm). (C) Comparison of protein condensation (data from Wallace et al.)^34^ and mRNA condensation (this study), and points show individual genes (protein or mRNA) (violin plot; median). (D) Transcript pSup decreases with length under all conditions, including in unstressed cells at 30°C. Stress induces additional shifts in sedimentation beyond this baseline. Each point represents a single gene’s transcripts at a given temperature, with the solid black line showing the mean pSup for transcripts of that length. *PMU1* mRNA, highlighted with a black border, sediments like a transcript more than three times its expected size following 42°C heat stress (dotted line). (E) A simple clustering model (see Methods S1) captures average pSup and stress-induced changes. Vertical boundaries mark the 1st and 99th percentiles of transcripts by length. (F) Sed-seq data allow quantification of key features: differential sedimentation relative to control (ΔSed) and escape from sedimentation (eSed). Both ΔSed and eSed control for effects of transcript length and are in units of σ, the standard deviation (SD) in sedimentation (dotted lines) around the length-dependent mean (solid lines); see STAR Methods. (G) Top: virtually all transcripts (gray points/density) sediment in response to 42°C heat shock, i.e., condensation, indicated by ΔSed > 0. Bottom: most genes (83%) in the heat shock factor 1 (Hsf1) regulon (orange points/density) show significant escape from sedimentation (eSed > 0). See also Figure S1.

We previously used sedimentation fractionation to isolate stress-induced protein condensates in yeast.^34,39^ To measure mRNA condensation, we coupled the same assay with RNA sequencing to develop Sed-seq (Figure 1A). Transcript abundances were quantified in total, supernatant, and pellet fractions, and the proportion of each mRNA in the supernatant (pSup) was calculated^34^ and validated by qPCR (Figure S1A). EDTA was added to dissociate polysomes that would otherwise co-sediment with condensates (Figure S1B).^34,40,41^

We first used Sed-seq to examine mRNA sedimentation in unstressed conditions (30°C) and after short heat shocks at 42°C and 46°C. As expected, 46°C produced SGs, visible as foci of poly(A)+ RNA marked by poly(A)-binding protein (Pab1), while the milder 42°C shock did not produce visible SGs (Figure 1B). Sed-seq revealed large decreases in pSup across the transcriptome during heat shock, correlated with the intensity of the stress just as in the case of proteins,^34^ which we interpret as stress-induced condensation. Unlike stress-triggered protein condensation of a minority of the proteome,^34^ virtually all transcripts show substantial condensation after stress (Figure 1C). Similar to protein condensation,^7,34,42^ mRNA condensation occurs at 42°C even when SGs are not apparent.

Long transcripts showed stronger sedimentation in all conditions, including in unstressed control cells (Figure 1D), underscoring the necessity of measuring differences between treatment and control to isolate condensation. Purified total mRNA from fission yeast spiked into unstressed lysate recapitulated this length effect, and spiked-in mRNA remained soluble in stressed lysate (Figure S1C), indicating that length-dependent sedimentation is separate from stress-induced condensation, which occurs prior to lysis. Indeed, a simple two-parameter physics-based model in which messenger ribonucleoproteins (mRNPs) sediment only due to their mass fits the average sedimentation of unstressed-cell transcripts well (Figures 1E, S1D and S1E; Methods S1), with substantial deviation only for the longest 1% of transcripts, which sediment less than predicted.

Stress-induced condensation of RNA shows little length dependence, and even short transcripts show a substantial increase in condensation after stress (Figure 1D). With two additional parameters reflecting stress-induced changes in the probability of inter-mRNA interactions per nucleotide (length-dependent) and per molecule (e.g., per 5′ or 3′ end, length-independent), the model fits the treatment averages closely, again deviating substantially only for the longest 1% of transcripts (Figure 1E). Length-independent interactions have stronger effects on condensation than length-dependent interactions for >99% of transcripts at 46°C (Methods S1), and a model without a length-independent parameter is sharply rejected for both treatments (Figure 1E, dotted lines; Figure S1E; *F* = 4,134.66 [42°C], *F* = 21,507.21 [46°C], *p* < 10^−6^ in each case; Methods S1). These results reveal a surprisingly minor role for interactions correlated with transcript length in promoting condensation.

We next derive an estimate of condensation per mRNA (Figure 1F; see STAR Methods). We calculate a differential sedimentation score (ΔSed), the length-controlled difference between treatment and control in units of σ, the standard deviation (SD) of unstressed control transcripts of similar length. An increase in ΔSed quantifies the treatment-dependent increase in sedimentation due to stress, which we interpret as condensation.

To quantify the escape from differential sedimentation (eSed), we score the difference between a particular transcript’s ΔSed and the mean ΔSed of transcripts of similar length (Figure 1G). Certain transcripts showed notably different changes in sedimentation and escape in response to stress relative to the rest of the transcriptome. For example, genes regulated by heat shock factor 1 (Hsf1), the master regulator of the core heat-shocked response, showed less sedimentation (ΔSed) and greater escape (eSed) (Figure 1G).

Our conclusions differ substantially from another report of the SG transcriptome in yeast, which concluded that transcripts accumulate in SGs in proportion to their length.^4^ In this prior study, sedimentation is the only means by which condensed material is enriched,^43^ justifying a direct comparison. We hypothesized that the prior study’s inability to correct for length-based sedimentation, due to lack of a non-stress control, created an artifactual enrichment for long transcripts. To match stress conditions, we treated cells with 0.5% azide or mock conditions and performed Sed-seq. ΔSed in these data and previously reported SG enrichment were anticorrelated (*r* = −0.3, *p* < 10^−6^) (Figure S1F). Our Sed-seq results on unstressed cells reproduce the previously reported results to a high degree of accuracy (*r* = 0.77, *p* < 10^−6^; Figure S1G) due to stress-independent sedimentation of long transcripts, not SG formation. Thus, controlling for mRNA length is necessary to avoid artifactual conclusions and to extract signals of biological regulation from sedimentation-derived data.

### Stress-induced mRNAs escape condensation and are preferentially translated

**Figure 2.**
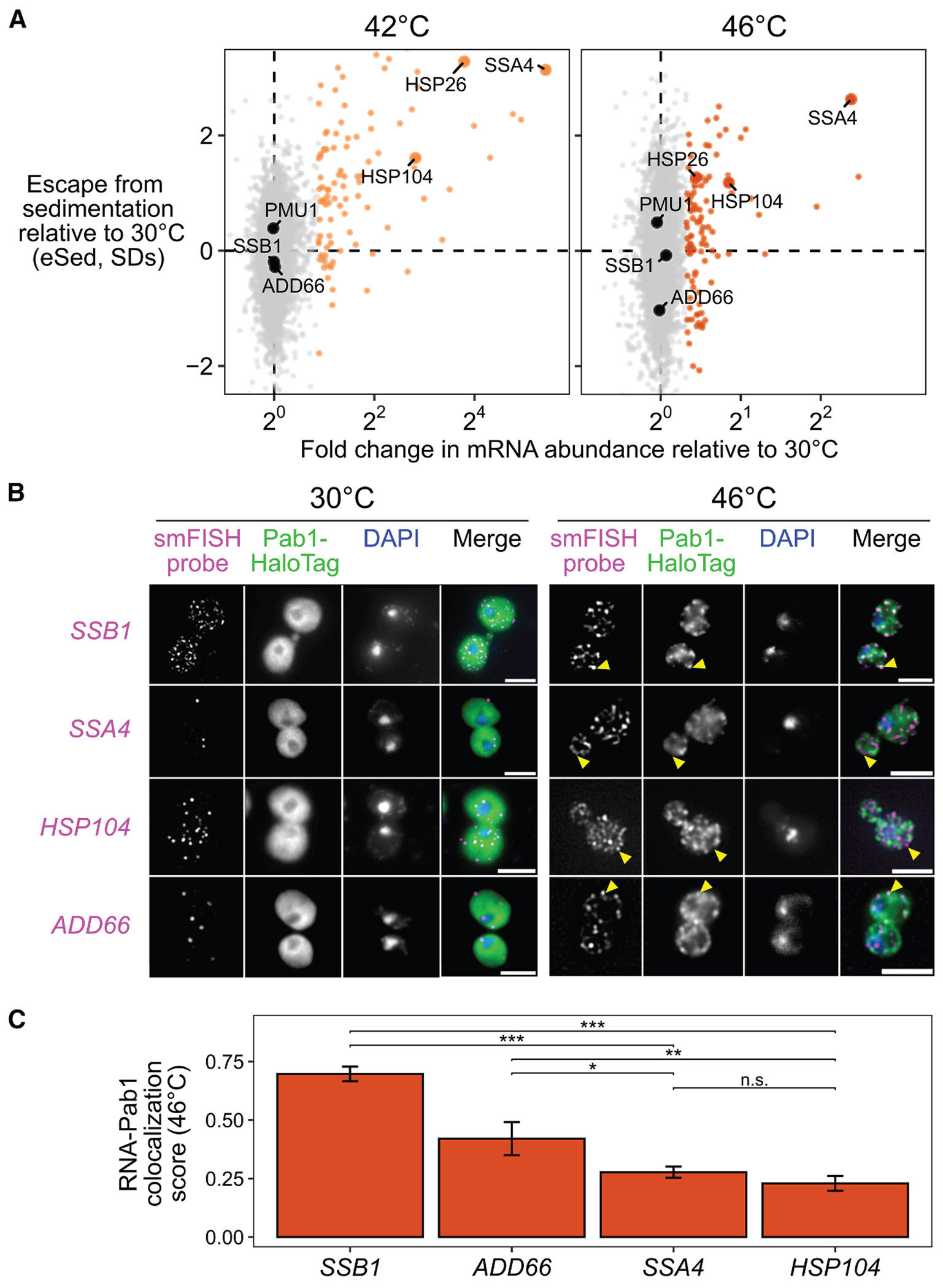
Induced transcripts escape condensation during heat shock (A) Comparison of mRNA abundance changes and escape from condensation (eSed) during heat shock reveals that transcript induction correlates with escape. In color are the top 100 most induced genes for each respective stress treatment. Labeled genes are mentioned elsewhere in the manuscript. (B) smFISH of induced (*SSA4/HSP104*) and uninduced (*SSB1/ADD66*) transcripts confirms that induced mRNA are not localized to Pab1-HaloTag marked SGs (scale bar, 5 μm). (C) Colocalization was quantified by comparing the intensity of the Pab1 channel in regions with mRNA foci to random regions in each cell (mean ± SE; see STAR Methods). See also Figure S2.

Transcripts strongly induced during stress consistently escaped condensation (Figure 2A). Targets of both Hsf1^44^ (as in Figure 1G) and Msn2/4^45^ showed positive eSed values, but only when highly induced, indicating that transcriptional activation—not transcription-factor identity—predicts escape (Figures S2A and S2B; Wilcoxon rank sum test *p* < 10^−6^).

To test whether these transcripts are also excluded from visible SGs, we used single-molecule fluorescence *in situ* hybridization (smFISH) to examine localization of paired transcript species. Constitutive Hsp70 *SSB1/2* transcripts colocalized with Pab1-marked granules after 46°C heat shock, whereas the induced Hsp70 *SSA4* transcripts were largely excluded (Figure 2B). Likewise, induced long *HSP104* (2,873 nt) transcripts were excluded, while uninduced short *ADD66* (896 nt) transcripts were recruited (Figure 2B). Quantification of the Pab1 signal confirmed strong enrichment for *SSB1* and *ADD66* but not for *SSA4* or *HSP104* (Figures 2C and S2C). Thus, together, Sed-seq and smFISH consistently show that stress-induced transcripts, independent of length, disproportionately escape stress-induced mRNP condensation.

**Figure 3.**
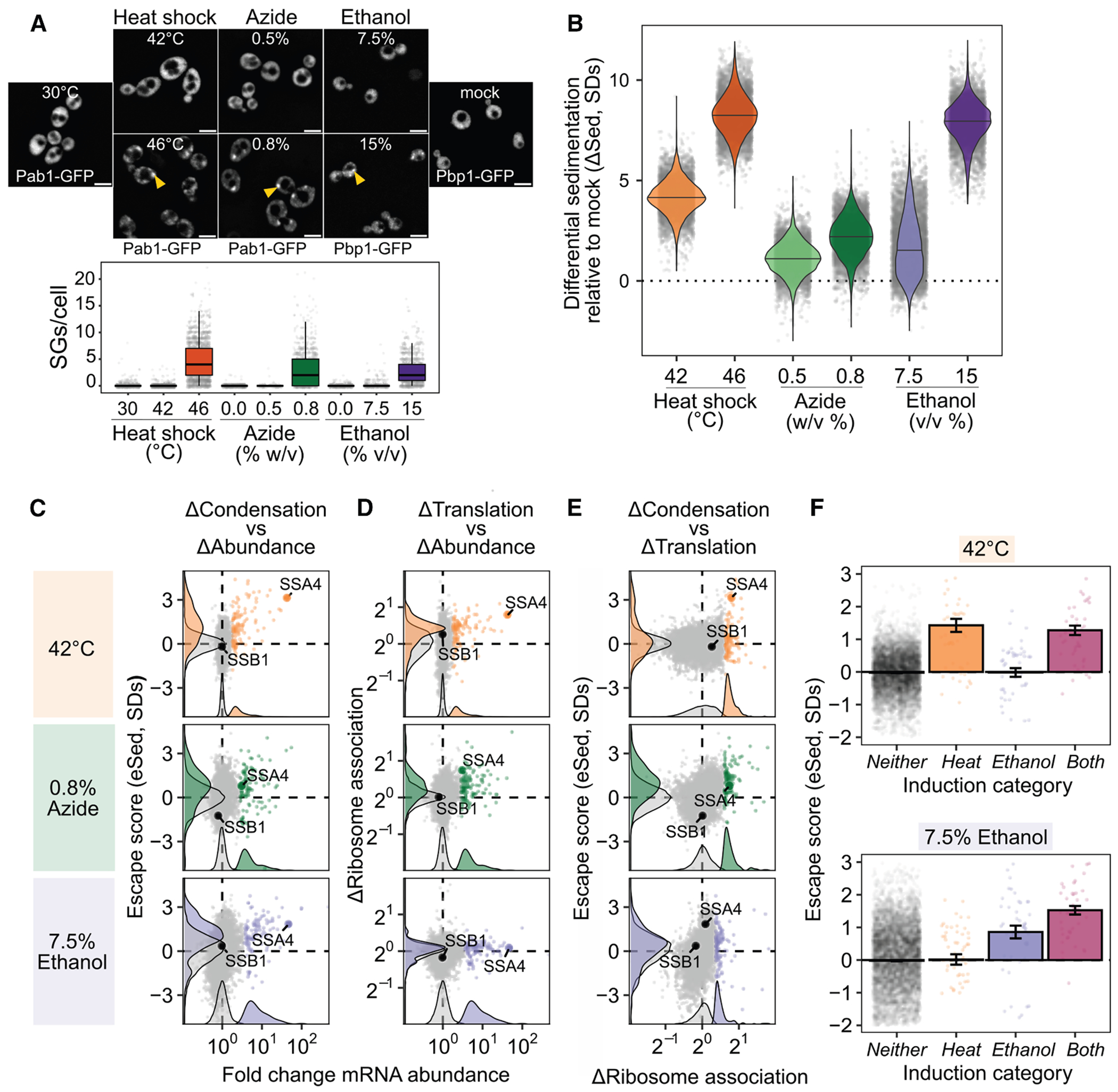
Newly transcribed and well-translated mRNAs escape condensation across stresses (A) Severe, but not mild, stress induces visible stress granules (SGs) across multiple conditions (scale bar, 5 μm). In the bottom panel, each point represents one cell (boxplot; median ± quartile). (B) Both mild and severe stress induce transcriptome-wide sedimentation of mRNA (ΔSed, units of standard deviations [SDs]), correlating with the severity of the stress. Each point represents one transcript species in this and subsequent panels (violin plot; median). (C) Across stresses, the most induced mRNA (the top 100 induced transcripts are highlighted) escapes from condensation (eSed). (D) Polysome-seq was used to measure the stress-induced change in ribosome association (the top 100 induced transcripts are highlighted). (E) Transcripts with increased translation tend to escape condensation in each stress (the top 100 translationally upregulated transcripts are highlighted). (F) The correlation between changes in translation and sedimentation is stress-specific. Transcripts from genes whose abundance increases during heat shock, but not ethanol stress, escape condensation during heat shock, but not ethanol stress, and vice versa (mean ± SE). See also Figure S3.

To test whether escape from condensation is heat shock specific, we performed Sed-seq on cells exposed to the common SG inducer sodium azide (NaN_3_)^4,43,46,47^ or to ethanol^48^ (Figure 3A). Using Pab1-GFP for heat and NaN_3_ and Pbp1-GFP for ethanol,^34,46,48^ we observed visible SGs only under severe stress, whereas transcriptome-wide condensation occurred under all conditions (Figure 3B). Condensation magnitude and variability differed across stresses but consistently increased with stress severity, with no enrichment for long transcripts (Figure S3A).

In a previously reported SG transcriptome of yeast exposed to azide stress, induced transcripts were not depleted (Figure S3B).^4^ By contrast, stress-induced transcripts escaped condensation in every stress tested (Figures 3C and S3C), confirming that this behavior is not limited to heat shock and echoing early results reporting exclusion of nascent transcripts from SGs.^12,13^ To assess coupling between translation and condensation, we measured ribosome association using polysome profiling followed by RNA sequencing (Polysome-seq).^49^ In heat and azide stress, but not ethanol stress, induced transcripts tended to be preferentially translated (Figures 3D and S3D), and across all conditions, preferential translation correlated with escape from condensation (Figures 3E and S3E). Thus, transcriptional induction, translation, and condensation are coordinated features of the stress response.

Finally, transcripts induced only in one stress escaped condensation only in that same stress (Figure 3F), indicating that escape is stress-specific and driven by the timing of transcript production rather than intrinsic sequence features.

### Transcript age and translation independently regulate condensation during stress

The escape of stress-induced mRNAs supports a new-transcript model, in which newly synthesized RNAs remain resistant to condensation for a short period during stress. This model predicts that escape scales with induction level, which is directly related to the fraction of transcripts that are newly made. A major alternative is that sequence-encoded features determine escape, which predicts escape independent of induction level. Sed-seq data support the new-transcript model: escape increases with induction (Figures 3C and S3C).

**Figure 4.**
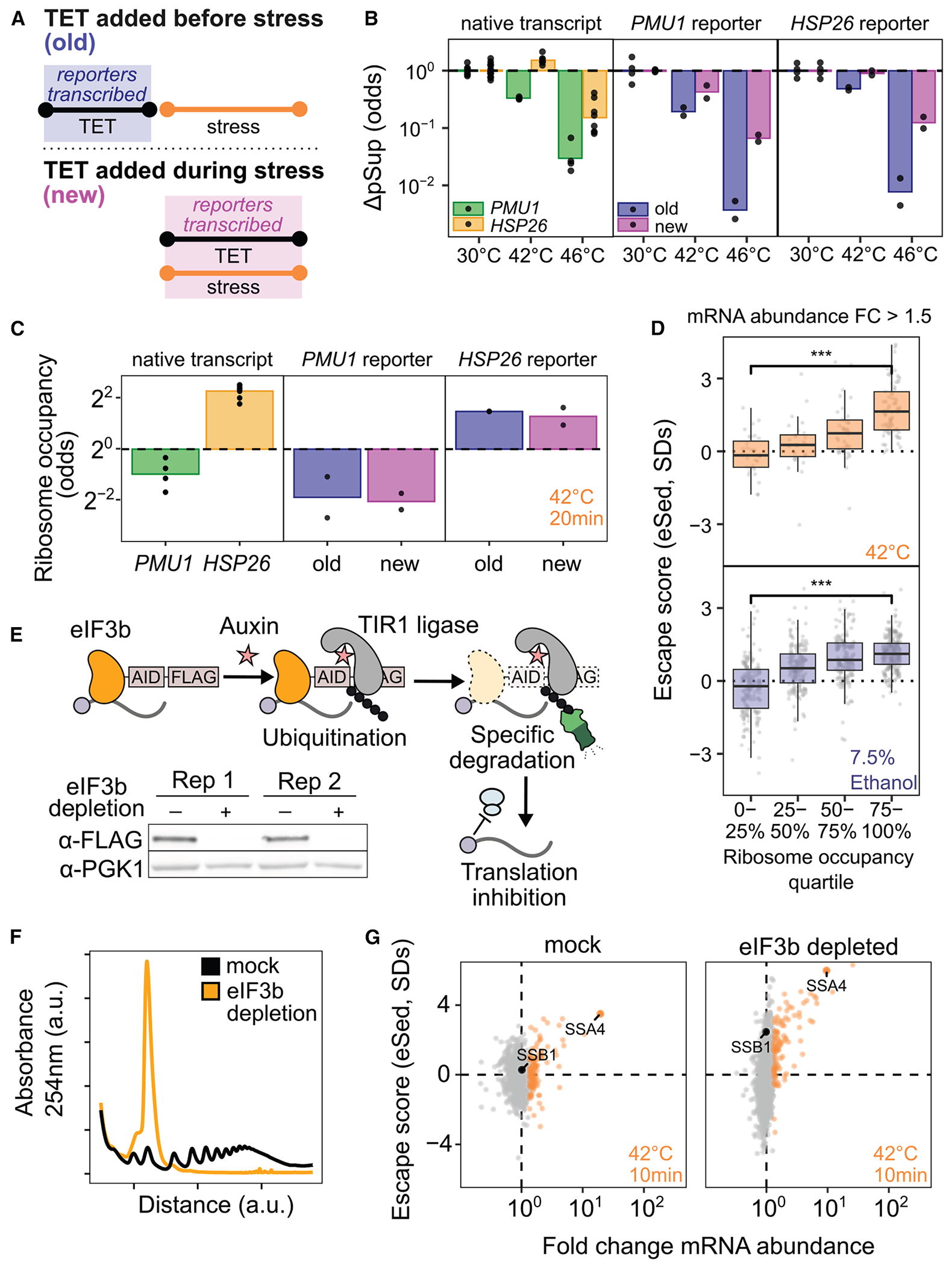
Translation and induction are independently sufficient to promote escape from condensation (A) TET-inducible reporter transcripts were generated with sequences derived from a stress-induced transcript (*HSP26*) or a stress-uninduced transcript (*PMU1*). (B) New transcripts sediment less than old transcripts for both reporters, as measured by centrifugation and qPCR after 10 min of stress (geometric mean). (C) The *HSP26*-derived reporter transcript is better translated than the *PMU1* reporter after 20 min of stress regardless of induction timing, as measured by RT-qPCR analysis of ribosome association using sucrose cushions (geometric mean). (D) Analysis of transcriptome-wide data in Figure 3 shows that even among stress-induced mRNAs, escape from condensation (eSed in standard deviations [SDs]) is correlated with ribosome occupancy (Wilcoxon rank sum test, N.S.: *p* ≥ 0.05, **: *p* < 0.01, ***: *p* < 0.001). Each point represents one transcript species (boxplot; median ± quartile). (E) The auxin-induced degradation system was used to deplete translation initiation factor eIF3b. Western blot has been cropped for clarity. (F) Depletion of eIF3b leads to translational collapse as measured by polysome profiles (*x* axis shows distance down the sucrose gradient). (G) Even in the absence of translation initiation, stress-induced transcripts still escape condensation after 10 min of 42°C stress. Each point represents a transcript species, with the top 100 induced transcripts per condition highlighted. See also Figure S4.

To test the effect of mRNA production timing directly, we built tetracycline (TET)-inducible reporters using the untranslated regions (UTRs) of heat-induced *HSP26* and heat-insensitive *PMU1* (Figure 1G). Each reporter was induced either before or during heat shock, and condensation was quantified by sedimentation and qPCR (Figures 4A and 4B). Both reporters were soluble at 30°C and condensed at 42°C and 46°C when expressed before stress. When induced during heat shock, however, both showed strongly reduced condensation (ANOVA *p* < 0.001). Thus, expression timing dictates condensation fate.

Ribosome-occupancy assays (Figures S4A–S4E)^50^ confirmed that translation differences do not explain this effect. After 20 min at 42°C, *HSP26* reporters had high ribosome occupancy, while *PMU1* reporters had low occupancy whether new or old (Figure 4C). Escape from condensation of new transcripts is therefore not a simple consequence of translation status.

While the reporters show that condensation can be altered independently of translation, the poorly translated *PMU1* reporters do condense more than the *HSP26* reporters (ANOVA *p* < 0.001). Transcriptome-wide, even among transcriptionally induced transcripts, poorly translated mRNAs do not escape condensation (Figure 4D). To further investigate the relationship between translation and condensation, we generated a strain of yeast with an auxin-inducible degron (AID) tag on the C terminus of eIF3b, a subunit of the essential initiation factor eIF3.^51,52^ Western blotting confirmed successful degradation (Figure 4E), which caused polysome collapse (Figure 4F). We then performed Sed-seq on samples heat-shocked after 2 h of mock treatment or eIF3b depletion. Even in cells with translation initiation blocked by eIF3b depletion, induced messages escape condensation (Figure 4G), indicating that active translation is not required for escape. Together, these results indicate that two features simultaneously contribute to escape from condensation: being newly transcribed during stress and being well-translated.

### TIICs of mRNA and protein precede SG formation and form in the absence of stress

What causes condensation? Translation initiation inhibition, by generating ribosome-free mRNA, is central to most models.^19^ We therefore quantified the sedimentation of each transcript within a condition relative to the smoothed mean of similar-length transcripts (rSed) again expressed in SD units (see STAR Methods).

**Figure 5.**
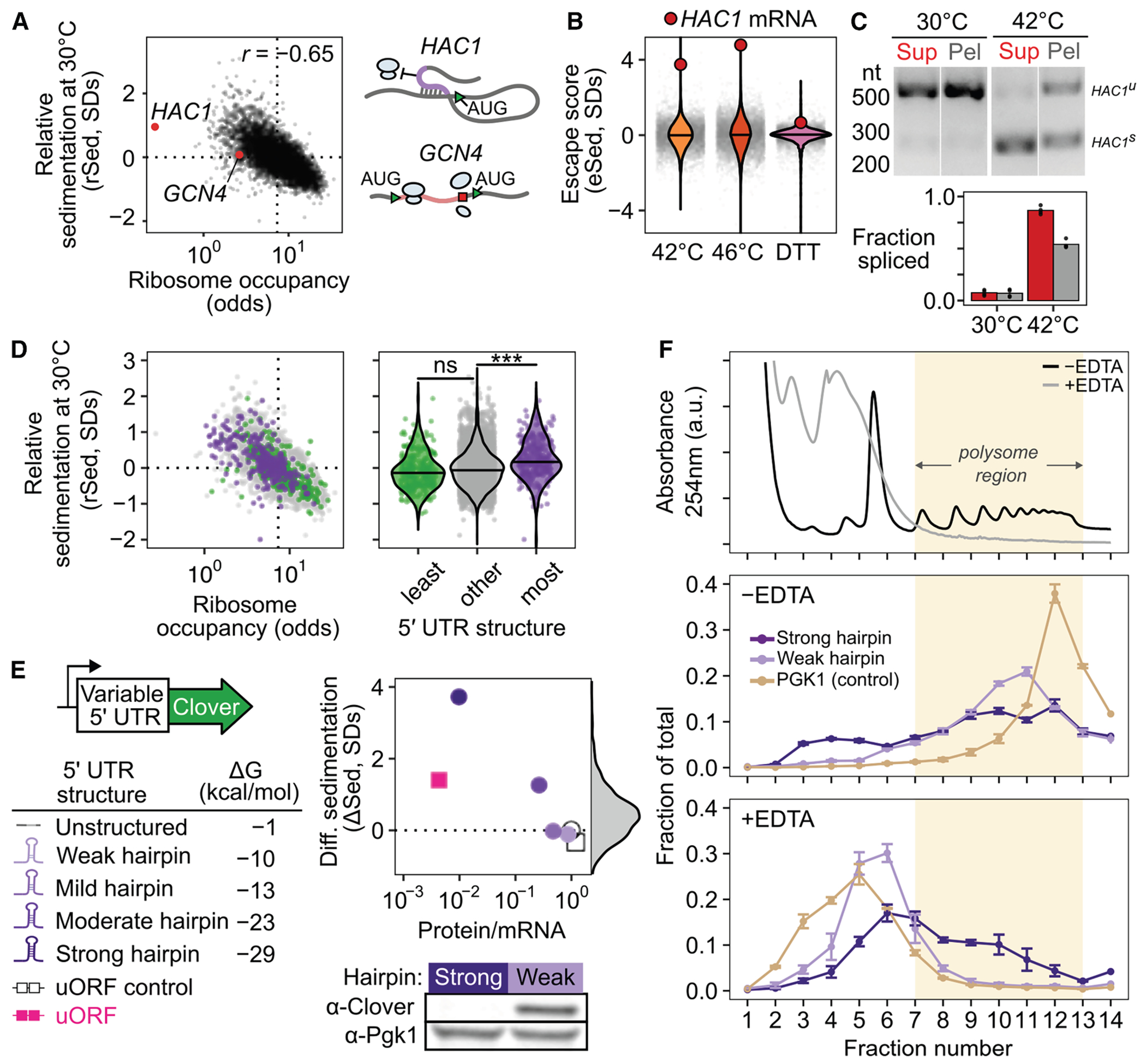
TIICs form in the absence of stress (A) Relative sedimentation (rSed in standard deviations [SDs] compared with the smoothed mean of similar-length transcripts) and translation measured by ribosome occupancy are negatively correlated in unstressed cells. Initiation-blocked *HAC1* shows strong sedimentation, but uORF-regulated *GCN4* does not. Each point represents a transcript. (B) *HAC1* mRNA becomes less condensed during heat shock and DTT treatment, as measured by escape from sedimentation. Transcriptome-wide eSed scores are shown for comparison (violin plot; median). (C) 42°C treatment leads to splicing of *HAC1* mRNA as measured by RT-PCR of supernatant (red) and pellet (gray) fractions. Both the spliced (*HAC1* ^*s*^) and unspliced (*HAC1*^*u*^) forms are present after treatment (mean). Gel has been cropped for clarity. Not shown: an unidentified band of ~1 kbp was present in all lanes. Original gel available upon request. (D) Left: ribosome occupancy in unstressed cells correlates well with length-normalized sedimentation. Right: the amount of computationally predicted structure in the 5′ UTR of transcripts predicts their relative sedimentation (violin plot; median). (E) Sedimentation reporters with variable 5′ UTRs were generated, which repress translation via either structured hairpin or uORF sequences. The protein/mRNA ratio was quantified by the normalized steady-state ratio of Clover abundance by flow cytometry to mRNA abundance by RNA-Seq (geometric mean). ΔSed was measured using Sed-seq on each reporter strain (mean). Western blot has been cropped for clarity. (F) EDTA disrupts ribosome association of *PGK1* and weak hairpin mRNAs but does not disrupt polysome-scale condensates containing initiation-blocked strong hairpin reporter mRNA (mean ± SE). See also Figure S5.

Surprisingly, even in untreated cells at 30°C, ribosome occupancy and relative sedimentation were inversely correlated (*r* = −0.65, *p* < 10^−6^; Figure 5A). Transcripts of *HAC1*, encoding the master regulator of the unfolded protein response (UPR), drew our attention given their high rSed and low occupancy. *HAC1* mRNA relies on a long-range base-pairing interaction between its 5′ UTR and unspliced intron to block translation initiation.^53^ By contrast, the other abundant mRNA in yeast that is translationally repressed in unstressed cells—*GCN4*, encoding the master regulator of the amino acid starvation response—initiates translation normally on upstream reading frames, preventing translation of the main coding region.^54^ Both mRNAs have similar lengths (*GCN4*: 1,465 nucleotides, *HAC1*: 1,197 nucleotides), and both are in the bottom 5% of all transcripts for ribosome occupancy (Figure 5A). Yet while *GCN4* has an rSed near the mean (rSed = −0.08, 45th percentile), *HAC1* sediments far more than the transcriptome average in unstressed cells (rSed = 0.95, 95th percentile) (Figure 5A).

During heat shock at 42°C and 46°C, *HAC1* mRNA showed strong escape from condensation (Figure 5B), despite showing no transcriptional induction (Figure S5A). The translation initiation inhibition of *HAC1* is relieved by mRNA splicing in the cytoplasm, leading to translation of the encoded Hac1 transcription factor, Hac1 nuclear import, and subsequent UPR activation.^53,55^ We hypothesized that heat shock at 42°C caused dissolution of condensates containing *HAC1* mRNA corresponding to relief of translation initiation inhibition by splicing. Multiple predictions follow: (1) *HAC1* TIIC dissolution should occur during activation by other UPR triggers, (2) *HAC1* should be spliced in response to the short heat shocks that trigger TIIC dissolution, and (3) if *HAC1* mRNA is translated, the resulting Hac1 transcription factor should drive transcription of UPR genes.

We tested each of these predictions in turn. First, we performed Sed-seq on cells treated with dithiothreitol (DTT), a standard UPR trigger. Confirming our prediction, *HAC1* mRNA showed among the strongest condensate escape across the entire transcriptome upon DTT treatment (Figure 5B) again despite showing no transcriptional induction (Figure S5A). Reductions in *HAC1* relative sedimentation accompanied increases in ribosome association across all stresses (Figure S5B). Second, we examined *HAC1* splicing in response to an 8-min, 42°C heat shock. Before shock, *HAC1* mRNA was unspliced, running as a single large band. After shock, the spliced form of *HAC1* appeared as a smaller band (Figure 5C), confirming our second prediction. Under these conditions, *HAC1* is incompletely spliced, and the spliced form of *HAC1* preferentially partitioned into the soluble fraction (Figure 5C). These observations are consistent with the release from condensates only of spliced *HAC1* transcripts in concert with their translational activation, while unspliced, initiation-blocked *HAC1* transcripts remain condensed.

Third, we looked for transcription of UPR genes^56^ at 42°C. We observed a slight but unmistakable induction after a 10-min 42°C shock (Figure S5C, Wilcoxon rank sum test *p* < 10^−6^). Next, we predicted that other heat-shocked data would show induction of the UPR at 42°C. Indeed, data from a systematic study of the heat-shocked response in budding yeast^57^ revealed that UPR targets were significantly induced by 10- or 30-min shocks at 42°C (Wilcoxon test *p* values < 10^−3^ in both cases) but not at 37°C (Wilcoxon test *p* = 0.15 and 0.70) (Figure S5D).

Together, these results support a simple and previously unappreciated sequence of events during *HAC1* activation: *HAC1* mRNA resides in initiation-blocked condensates under basal conditions and is spliced and released from condensates upon UPR-inducing stress, coinciding with translation of the Hac1 transcription factor protein, which then drives UPR regulon transcription.

More broadly, *HAC1* mRNA condensates appear to be an extreme example of a transcriptome-scale phenomenon linking reduction in translation with condensation (Figure 5A). Lack of condensation by upstream open reading frame (uORF)-regulated, freely initiating *GCN4* mRNA suggests that a block in initiation, rather than ribosome-free mRNA, is the key correlate of condensation. Consistent with this, transcripts with the most predicted structure in their 5′ UTR, inversely correlated with initiation efficiency,^58^ had higher rSed than the bulk transcriptome (Figure 5D).

These results provide evidence that, transcriptome-wide, mRNA inhibited in translation initiation is found in condensates, even in unstressed cells. Anticipating later results indicating these condensates are distinct from previously described bodies, we refer to them as translation-initiation-inhibited condensates (TIICs, pronounced “ticks”).

Does inhibition of translation initiation cause TIIC formation? We asked whether we could recapitulate *in vivo* endogenous transcript-specific condensation using a series of synthetic mRNAs encoding the green fluorescent protein Clover, with progressively stronger translation initiation blocks created by hairpins in their 5′ UTR.^59^ These hairpin series blocked translation initiation, as measured by the ratio of fluorescence intensity to mRNA abundance, with more stable hairpins more completely blocking translation (Figure 5E). Western blotting against Clover confirmed translation was permitted by the weak hairpin and blocked by the strong hairpin (Figure 5E).

As predicted, these constructs exhibited increased sedimentation inversely correlated with their translation, mirroring *HAC1* mRNA. By contrast, a synthetic uORF construct built from the *GCN4* 5′ UTR yielded substantially less condensation relative to a disrupted-uORF control^60^ than the most stable hairpin construct, despite showing stronger translational repression (Figure 5E). These experiments demonstrate that even in unstressed cells, translation initiation inhibition causes mRNA condensation, producing TIICs.

### TIICs are polysome-scale mRNP condensates

To further characterize TIICs, we performed polysome profiling, separating mRNP species by size on a sucrose gradient, followed by qPCR on strains expressing strong or weak hairpin reporters (Figure 5F). The weak hairpin reporter transcript, along with the endogenous housekeeping transcript *PGK1*, co-sedimented with the heavy polysome-associated fractions, consistent with active translation and our western blot data. Strikingly, the strong hairpin reporter transcript also co-sedimented with heavy polysome fractions despite being translationally repressed.

To determine whether strong hairpin sedimentation was due to TIIC formation rather than residual ribosome association, we dissolved ribosomes and polysomes by treating lysate with EDTA prior to sedimentation. As expected, the weak hairpin and *PGK1* transcripts shifted to lighter fractions, consistent with loss of ribosome association. By contrast, the strong hairpin transcript remained in heavy fractions, demonstrating that TIICs are resistant to EDTA treatment and sediment based on size rather than ribosomal binding (Figure 5F).

To test whether TIICs pellet in heavy fractions due to membrane association, we performed a membrane flotation assay (Figure S5E).^61^ The strong hairpin transcript was detected in bottom fractions regardless of the presence of membrane-dissolving Triton X-100, similar to *PGK1* and *TUB2* transcripts (Figure S5F), indicating that TIIC sedimentation is not due to membrane association. This is supported by transcriptome-wide analysis of relative sedimentation, which shows that transcripts encoding secreted proteins do not show increased sedimentation (Wilcoxon rank sum test *p* > 0.99, Figure S5G).^62^

Together, these results further confirm the identification of TIICs as mRNP condensates, unassociated with membranes, which appear in a range of sizes up to several megadaltons (see Methods S1).

### Blocking translation initiation at distinct steps causes global mRNA condensation and implicates an upstream, competitive step

**Figure 6.**
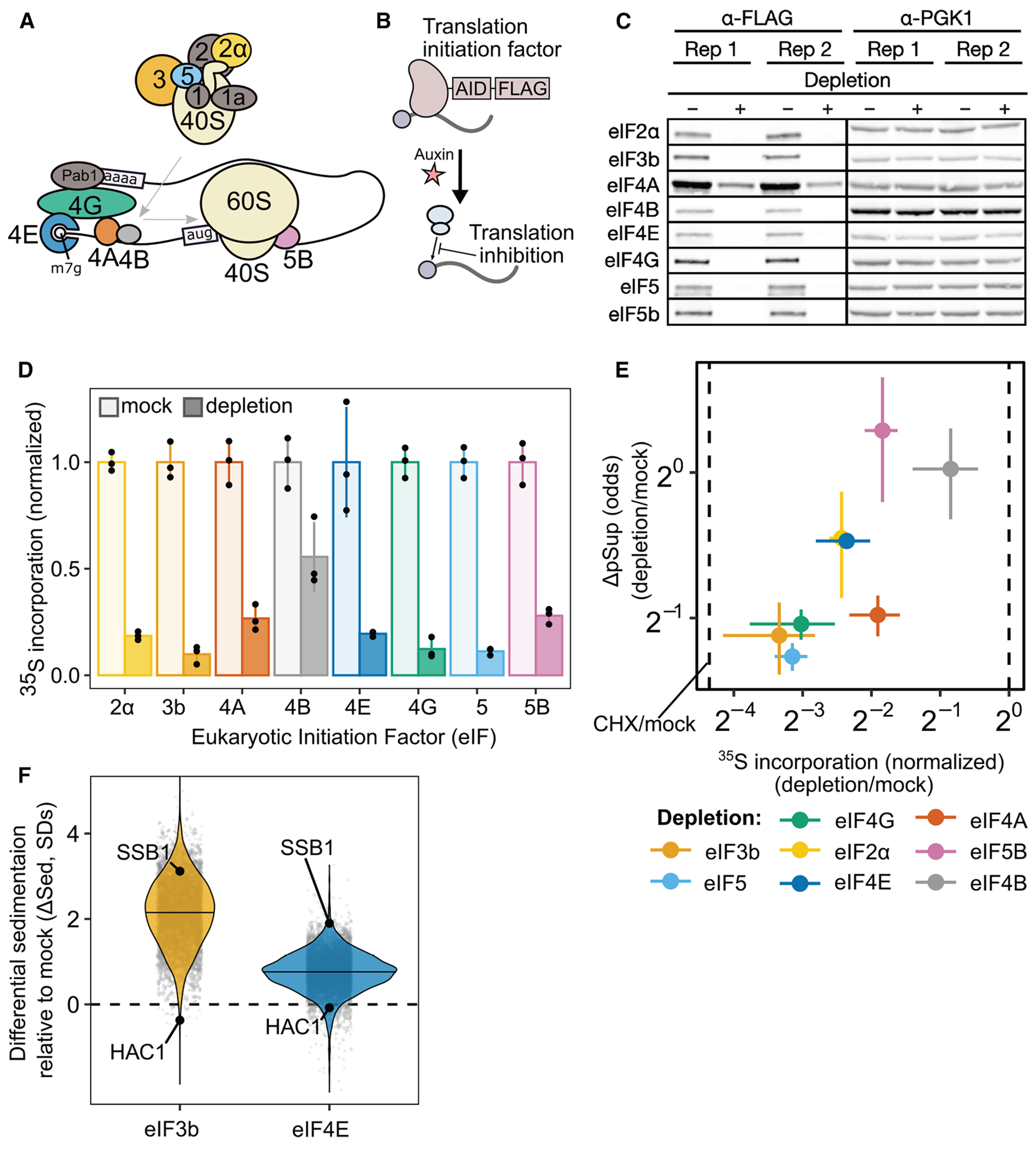
Global translational initiation inhibition triggers transcriptome-wide TIICs (A and B) (A) Translation initiation factors involved in various steps of initiation were (B) depleted via the auxin-inducible degradation system. (C) Depletion for each factor was verified via western blot with Pgk1 used as a control. Western blot has been cropped for clarity. (D) Inhibition of global translation caused by depleting each initiation factor was measured by the incorporation of radiolabeled amino acids (mean ± SD). (E) The change in proportion in supernatant (ΔpSup) of *PGK1* and *BEM2* transcripts (mean of the two is plotted ± SE) correlates with the amount of translation block caused by each initiation factor depletion (mean ± SD). (F) Sed-seq was used after eIF3b and eIF4E depletion to measure mRNA sedimentation (violin plot; median). Depletion of both factors, and especially eIF3b, triggers global condensation. See also Figure S6.

To examine the results of global initiation blockade, we generated yeast strains with AID tags on eight eukaryotic initiation factors (eIFs) acting at multiple steps (Figures 6A–6C).^51,52^ eIF depletion resulted in polysome collapse (Figure S6A) and proteome-wide reduction in translation activity (Figures 6D and S6B–S6D).

We used qPCR to quantify the average pSup of two transcripts, *PGK1* and *BEM2*, following 2 h of initiation factor depletion. Blocking initiation caused mRNA condensation in each case, with the degree of blockade correlated with the extent of condensation (Figure 6E).

During global translation initiation block, we expect that all transcript species will form TIICs, leading to increased mRNA sedimentation transcriptome-wide. Using Sed-seq on strains depleted for eIF4E or for eIF3b, we observed transcriptome-scale mRNA condensation in both cases, to a profound degree after eIF3b depletion (Figure 6F). Because basally initiation-repressed mRNAs already enter TIICs in untreated cells, we predicted that they would show the smallest differences in sedimentation after eIF depletion. Consistent with this prediction, initiation-inhibited *HAC1* mRNA showed almost no change after both eIF depletions, whereas initiation-competent *SSB1* mRNA condensed (Figure 6F).

Together, these results show that blocking translation initiation globally triggers global mRNP condensation. We next sought to understand the relationship between TIICs, stress-induced mRNP condensation, and SGs.

### TIICs are likely SG precursors

**Figure 7.**
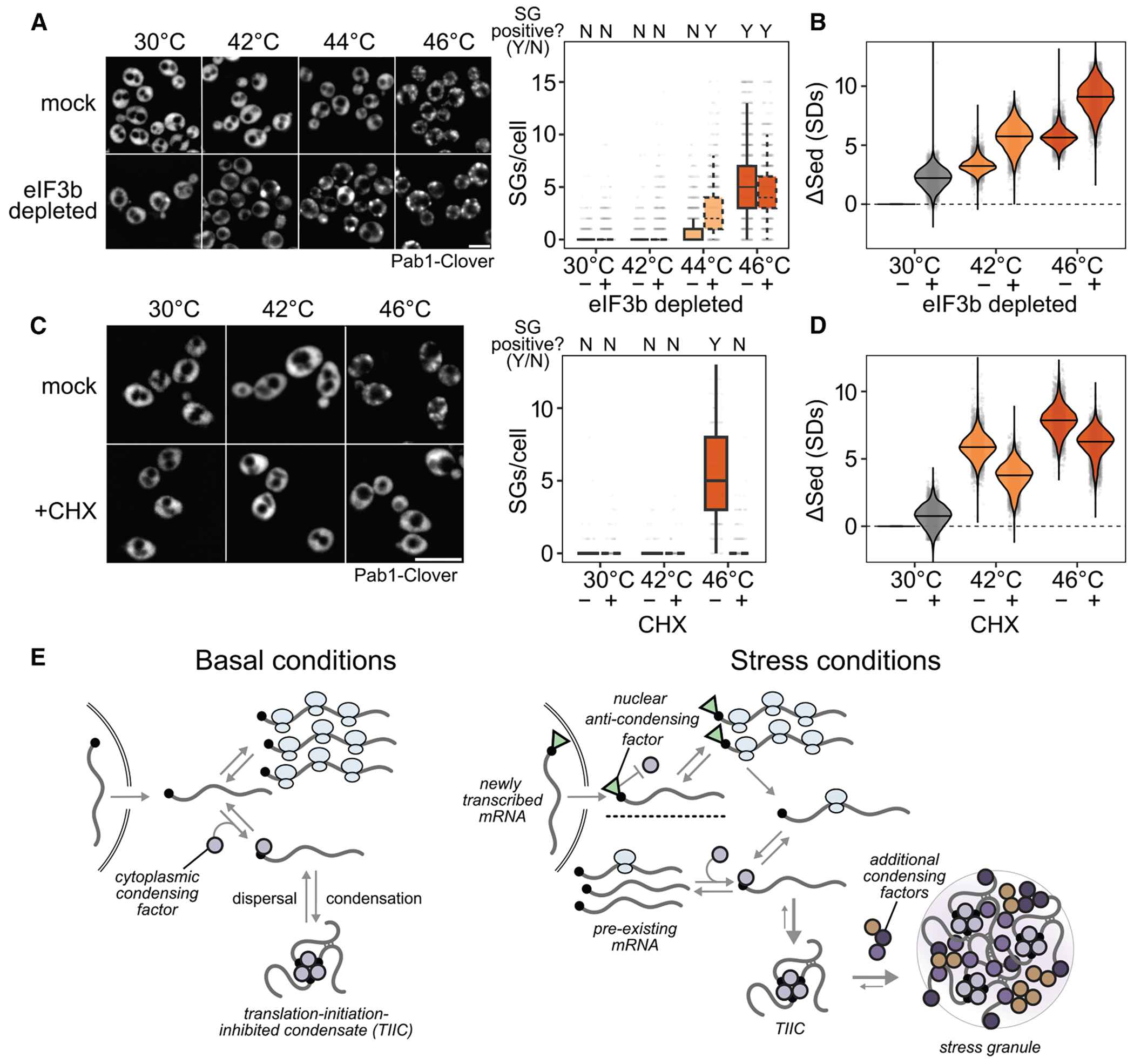
TIICs precede and potentiate SGs (A) SGs are potentiated in eIF3b-depleted cells where translation initiation is inhibited, shown by the appearance and penetrance of Pab1-Clover-marked SGs at lower temperatures compared with mock-treated cells (scale bar, 5 μm). Right: quantification of the presence of SGs in all conditions (boxplot; median ± quartile). (B) Sed-seq data comparing global condensation in eIF3b-depleted and mock cells after 2 h of depletion followed by 10 min of heat shock. eIF3b depletion triggers more RNA condensation at each temperature. Each point represents one transcript species (violin plot; median). (C) SG formation is prevented by inhibiting translation elongation with cycloheximide (CHX) (scale bar, 5 μm). Right: quantification of the presence of SGs in all conditions (boxplot; median ± quartile). (D) CHX treatment reduces, but does not prevent, stress-induced RNA condensation. Each point represents one transcript species (violin plot; median). (E) Model of the competition between translation initiation and TIIC formation during normal growth and stress. Well-translated transcripts are protected from condensation by competition between translation initiation and TIIC formation. During stress, newly transcribed transcripts escape stress-induced condensation, likely due to a 5′ bound protein or modification that inhibits condensation. In addition, global inhibition of translation leads to transcriptome-wide TIIC formation. These TIICs are precursors of visible SGs, whose formation involves additional stress-induced condensing factors.

We counted SGs before and after inhibiting translation initiation by eIF3b depletion, both in otherwise untreated and in heat-shocked cells (Figure 7A; see STAR Methods). After eIF3b depletion at 30°C, which causes substantial transcriptome-wide mRNP condensation (Figure 7B), cells are SG-negative (Figure 7A). We conclude that inhibiting translation initiation by eIF3b depletion causes TIIC formation but not SG formation.

Upon heat shock at 44°C, otherwise untreated cells are SG-negative, but when eIF3b is depleted, cells become SG-positive (Figure 7A). Thus, eIF3b depletion potentiates SG formation.

In every case, heat stress amplifies the sedimentation induced by translation initiation depletion (Figure 7B), suggesting that stress triggers additional condensation processes beyond translation initiation blockade alone.

We then asked how blocking SG formation affects mRNP condensation. Treatment with CHX prevents SG formation,^34,46,48^ which we confirm at 46°C heat shock (Figure 7C). However, CHX treatment sufficient to prevent SG formation reduces mRNP condensation only slightly (Figure 7D), consistent with the persistence of TIICs. Notably, CHX also disrupts processing bodies (P bodies),^63,64^ indicating that TIICs are not P bodies.

These results place TIICs upstream of SGs, preceding and potentiating their formation. Inhibition of translation initiation causes TIIC formation but not SG formation. Together, these results suggest that TIICs are building blocks for SGs.

## DISCUSSION

What is the physiological role of mRNP condensation in and outside of stress? Which mRNPs condense during stress, and why? What is the relationship between mRNP condensation, its functional causes and consequences, and SG formation?

We find that, across multiple stress conditions, preexisting mRNAs enter translationally silent condensates to a degree that depends on stress intensity. At the same time, stress-induced transcripts escape condensation and are robustly translated. The timing of transcript production, rather than any particular transcript feature, is a primary determinant of escape from condensation, which then permits selective translation. An important result from our study is that microscopically visible SGs play little if any role in these processes.

### Small mRNP condensates are pervasive in the absence of stress or SGs

Using a range of approaches, we discover pervasive mRNP condensation in cells without SG formation, and even in the absence of any discernible stress. Here, our results indicate submicroscopic molecular organization governed by translation initiation: initiation-blocked transcripts are recruited to subdiffraction-scale condensates. These TIICs can be generated individually by blocking message-specific initiation or at the transcriptome scale by blocking initiation, and they do not require environmental stress for their formation, and they can form when SGs are either absent or pharmacologically blocked. In short, TIICs are not SGs.

A range of stress conditions—physiological stresses such as 42°C heat shock and 5% ethanol, and the widely used 0.5% sodium azide—do not produce SGs in our hands but do produce mRNP condensation and the escape of new mRNAs. Thus, considerable biology would be overlooked by focusing only on SG-forming conditions.

### mRNP condensation in cells is not primarily driven by ribosome-free RNA

SGs have long been thought to form after translation inhibition and ribosome runoff, exposing ribosome-free RNA, which serves as a platform for new intermolecular interactions, whether directly between RNAs or mediated by RNA-binding proteins.^4,65–67^ This model could explain results showing stronger recruitment of longer mRNAs and the ability of bound ribosomes to prevent SG recruitment.^68,69^ We find that the recruitment of initiation-blocked mRNPs into TIICs prior to, and independent from, SG formation proceeds differently. Length has little effect. Two abundant mRNAs, *HAC1* and *GCN4*, both with long stretches of ribosome-free mRNA, show divergent behavior: initiation-blocked *HAC1* mRNA condenses, while uORF-regulated *GCN4* mRNA remains largely uncondensed. We reproduce these behaviors using synthetic mRNAs, isolating the critical role of blocked initiation rather than ribosome runoff for TIIC formation. Accordingly, locking ribosomes on mRNAs using CHX does not prevent TIIC formation.

Two models could explain why ribosomes prevent recruitment of mRNA to SGs. In the first model, ribosomes occlude naked mRNA that would otherwise cause recruitment to SGs. In the second, ribosomes inhibit the processes that recruit mRNAs to SGs, and naked mRNA plays little or no role. Falsifying the ribosome-occlusion model, we show that global translation initiation blockade and subsequent ribosome runoff from virtually all transcripts do not cause SG formation. Consistent with the inhibitory ribosome model, in mammalian cells, the presence of a single ribosome on an mRNA is sufficient to prevent SG recruitment even when the coding sequence is ribosome-free.^68^

Overall, ribosome-free mRNA appears to play at best a minor role in the formation of TIICs and SGs.

### A protein-mediated 5′ -cap-competition model coherently explains multiple mRNP condensate phenomena

Instead, we propose that competition for the mRNA 5′ cap by translation initiation and TIIC recruitment can explain all of our observations (Figure 7E). This model posits the (presumably protein-mediated) recruitment of mRNAs to TIICs via binding to free 5′ cap. Such a model explains why mRNA length has little influence on TIIC formation, as well as why ribosomes on the mRNA body do not disperse TIICs. Any process that blocks access to direct cap binding would interfere with TIICs in this model. For most mRNAs, binding of the cytosolic eIF4F complex containing cap-binding protein eIF4E is transient, stabilized by mRNA activation and translation initiation.^70^ Consequently, most mRNAs will transiently have unprotected caps, making them substrates for condensation, whereas stable protection will be conferred by translation initiation. This cap-competition model would explain why we consistently observe tradeoffs between initiation and condensation, whether at steady state, for synthetic constructs, or when initiation is blocked by stress or depletion of initiation factors.

### How do newly synthesized mRNAs escape condensates?

The cap-competition model hints at a mechanism by which newly synthesized mRNAs could escape condensation. Our transcriptomic and reporter assays show that transcripts transcribed during stress escape condensation regardless of sequence-encoded mRNA features or regulation by particular transcription factors. Consistent with our conclusion that timing is the key variable, an independent study of glucose withdrawal also shows that expression timing, rather than sequence, determines whether mRNAs escape stress-induced translational repression.^17^

One possible explanation for the role of timing is that new transcripts may be marked in some way before or during nuclear export, thus blocking condensation while permitting translation initiation. Translation is not required for exclusion of new transcripts, because even when translation is inhibited by depletion of eIF3b, newly transcribed transcripts still escape. What might this condensation-inhibiting mark be? Possibilities include changes in an mRNA modification such as methylation, in polyadenylation, or in binding of a protein factor. Notably, most capped mRNAs are protected by a nuclear cap-binding complex after transcription and during export. This complex may be stabilized in the cytoplasm during stress instead of being exchanged during a pioneer round of translation, and indeed nuclear cap-binding proteins can support active translation during stress.^71^ Such a complex would prevent cap-dependent condensation, thus privileging newly synthesized mRNAs.

### What are the functions of mRNP condensation?

An accounting of the cellular function of mRNP condensation must now contend with four facts: the presence of condensation in unstressed cells, the strong causal link to translation initiation inhibition, the weak dependence on mRNA length or sequence, and the exclusion of stress-induced messages. Exclusion of new messages and condensation of older messages favor an adaptive interpretation: stress-induced mRNP condensation helps cells to redirect translational activity toward transcripts most relevant to the cell’s current situation. This functional interpretation contrasts with previous models arguing that some RNA condensates are incidental byproducts^20^ or even for an “RNA entanglement catastrophe” following release of untranslated mRNA.^72,73^

We hypothesize that mRNP condensation provides cells with regulatory control over the translationally active transcriptome through a simple mechanism: preventing reinitiation of ribosomes on translationally stalled mRNAs by sequestering their 5′ ends. Condensation, by sequestering transcripts away from competitive processes such as decapping or reinitiation,^74^ temporarily stores these mRNAs for short-term retrieval by dispersal factors including molecular chaperones.^34,75^ Blocking reinitiation is crucial for redirecting translational activity and separable from another effect, which is implied: protection of mRNAs from degradation,^27,76,77^ another mechanism to prevent reinitiation. No part of this regulatory model requires the formation of visible SGs or other large membraneless organelles.

Beyond separating mRNP condensation from SG formation, a key advance reported here is to separate mRNP condensation from stress itself. How TIICs form, dissolve, and influence regulation outside of stress must now become a focus.

### Limitations of the study

Our study leaves open the molecular mechanisms for TIIC formation and the escape of new transcripts, as well as the composition and material state of TIICs. We present experimental methods for isolation and analysis of mRNP condensates, which involve differential sedimentation of EDTA-treated lysate. EDTA treatment, which prevents polysomal contamination of sedimented material, may also disrupt condensation or related processes that depend on magnesium or calcium. Direct visualization of TIICs remains to be achieved. Although we prefer a model where TIICs are precursors of SGs, future work studying the precise relationship between TIICs and SGs will be important. Finally, the phenomena reported here in budding yeast may differ in mammalian or other cells, warranting caution when generalizing.

## RESOURCE AVAILABILITY

### Lead contact

Further information and requests for resources and reagents should be directed to and will be fulfilled by the lead contact, D. Allan Drummond (dadrummond@uchicago.edu).

### Materials availability

All materials will be made available upon request.

### Data and code availability

- All raw sequencing data generated for this project have been deposited in GEO under accession code GEO: GSE265963. Raw imaging data are available at Mendeley (doi: 10.17632/hby6drmgy9.1). All other data are deposited at https://github.com/drummondlab/RNACondensation2025/ (doi: https://doi.org/10.5281/zenodo.15635226). These data are publicly available as of the date of publication.
- All original code is deposited at https://github.com/drummondlab/RNACondensation2025/ (doi: https://doi.org/10.5281/zenodo.15635226) and is publicly available as of the date of publication.
- Any additional information required to reanalyze the data reported in this paper is available from the lead contact upon request.

## STAR★METHODS

### EXPERIMENTAL MODEL AND STUDY PARTICIPANT DETAILS

#### Yeast

Unless otherwise stated, *Saccharomyces cerevisiae* BY4741 and BY4742 strains were grown in synthetic complete dextrose media (SCD), and exponentially growing cultures were incubated at 30°C for >12 hours until OD_600_ = 0.4 before exposing to stress for experiments.

### METHOD DETAILS

#### Cell growth and stress conditions

Unless otherwise noted, the BY4741 strain of *Saccharomyces cerevisiae* was used in experiments. All experiments were done with at least two biological replicates, starting from growth. Cells were grown at 30°C in synthetic complete dextrose media (SCD) for at least 12 hours to OD_600_ = 0.4 before being exposed to stress. Temperature stresses for sedimentation experiments were completed by centrifuging the culture and exposing the yeast pellet to either 42°C or 46°C water baths. Control cells were placed inside a 30°C incubator. Cycloheximide-treated cells were pre-treated for 10 minutes with 100 μg/mL cycloheximide (Sigma #C7698-5G) before heat shock. Azide stresses were completed at either 0.5% w/v or 0.8% w/v for 30 min in SCD adjusted to pH 6.8 with NaOH. Azide was added from a 10% w/v sodium azide stock in water. Mock treatments were completed by adding pure water at the same volume to cultures. Ethanol stresses were completed by resuspending centrifuged cell pellets in SCD made with either 5%, 7.5%, 10%, or 15% ethanol for 15 min. Control cells were mock treated by resuspending in normal SCD. DTT treated cells were treated with 10 mM DTT for 15 minutes prior to harvesting. Temperature stresses for polysome sequencing and for tet-inducible reporter experiments were done by growing 250 mL of yeast in SCD overnight to OD_600_ = 0.4, collecting yeast via vacuum filtration onto a 0.45 μm filter (Cytiva 60206), putting the filter in 125 mL of pre-warmed media and incubating in a temperature controlled shaking water bath or incubator. After the indicated time, samples were harvested again via vacuum filtration and immediately scraped into liquid nitrogen.

Yeast transformations were performed either using a standard lithium acetate transformation or Zymo Frozen-EZ Yeast Transformation II Kit (Zymo #T2001) before plating on appropriate selection media.^88^ Clones were verified by colony PCR and Sanger sequencing.

#### Generation of spike-in RNA

In-vitro transcribed (IVT) RNA or purified *Schizosaccharomyces pombe* total RNA was used as spike-ins where noted. The IVT RNA was produced by first amplifying a linear DNA fragment encoding NanoLuc using Q5 polymerase (NEB #M0494S), and purifying the DNA using an NEB clean and concentrate kit. The RNA was then made using a T7 Highscribe kit (NEB #E2040S), treated with DNase I (NEB #M0303L) and purified using an NEB clean and concentrate kit (NEB #T2030).

For the *S. pombe* RNA, fission yeast (FY527) was grown in YES media (5 g/L yeast extract, 30 g/L glucose, 225 mg/L adenine, histidine, leucine, uracil and lysine hydrochloride) at 32°C until OD_600_ = 0.5, harvested by centrifugation (3 minutes at 2500 g), resuspended in Trizol, and lysed by vortexing with 0.5 mm zirconia glass beads before extracting RNA using Zymo Direct-zol kits (Zymo #R2072).

#### Fractionation-by-sedimentation sequencing (Sed-seq)

Biochemical fractionation was completed similarly to Wallace et al.,^34^ with the major exception that 20,000 g for 10 min was used rather than the original 100,000 g for 20 min. In short, 50 mL cultures of treated yeast were harvested by centrifugation at 3000 g for 5 minutes, then resuspended in 100 μL of soluble protein buffer (SPB: 20 mM HEPES, pH 7.4, 140 mM KCl, 2 mM EDTA, 0.1 mM TCEP, 1:200 protease inhibitor (Millipore #539136), 1:1000 SUPERase·In RNase Inhibitor (Invitrogen #AM2696), and flash frozen in liquid nitrogen as a pellet in a 2 mL Eppendorf Safe-Lock tube (Eppendorf #0030123620) with a 7 mm steel ball (Retsch #05.368.0035). The cells were then lysed using a Retsch MM400 for 5x90s at 30 Hz, chilling in liquid nitrogen between each shaking repeat. The lysed cells were resuspended in 600 μL of SPB, and 100 μL of total sample was transferred to 300 μL of Trizol LS (Invitrogen #10296010). For the S. pombe spike-in experiment, purified S. pombe total RNA was added to the lysate immediately after resuspension in SPB. The remainder was centrifuged for 30 seconds at 3000 g, and 300 μL of clarified lysate was transferred to a new 1.5 mL tube. This was then centrifuged for 10 minutes at 20,000 g. A 100 μL supernatant sample was transferred to 300 μL of Trizol LS, and 400 μL of SPB was added to the pellet as a wash. After another spin at 20,000 g for 10 minutes, the supernatant was removed and the pellet was resuspended by vortexing for 15 minutes in 300 μL of Trizol LS and 100 μL of water. If required, 1 ng of spike-in transcript was added to each sample at this step before RNA was isolated using Zymo Direct-Zol RNA extraction columns (Zymo #R2052), and RNA integrity was assessed by the appearance of two sharp rRNA bands on a 1% agarose gel and quantified using the absorbance at 260 nm.

#### RNA quantification by RT-qPCR

Reverse transcription for qPCR was either performed using gene-specific reverse priming with the iScript^™^ Select cDNA Synthesis Kit (Bio-Rad #1708897) or using NEB LunaScript RT SuperMix kit (NEB #E3010L). In both cases, manufacturer protocols were followed using an input of 2.5 ng of RNA per μL of reaction. For gene-specific priming, the reverse primer was used at 5 μM. The IDT Primetime gene expression master mix (IDT #1055771) was used for quantitative PCR on a Bio-Rad CFX384 instrument with Taqman probes (1.5 μM for primers; 600 nM probe). For samples with spike-ins, abundances were calculated relative to the spike-in abundance using the ΔΔCq method.

#### Polysome collection and analysis

Around 100 mg of frozen yeast that was collected by vacuum filtration, or following centrifugation at 3,000g for 1 minute, was transferred to a pre-chilled 2 ml Eppendorf “Safe-Lock” tube. Cells were lysed with a pre-chilled 7 mM stainless steel ball (Retsch #05.368.0035) by 5x90sx30Hz pulses in a Retsch MM100 mixer mill, chilling in liquid nitrogen (LN2) between pulses. Sample was resuspended in 10:1 (v/w) polysome lysis buffer (20 mM HEPES-KOH (pH 7.4), 100 mM KCl, 5 mM MgCl2, 200 μg/mL heparin (Sigma #H3149), 1% triton X-100, 0.5 mM TCEP (Goldbio #TCEP25), 100 μg/mL cycloheximide (Sigma #C7698-5G), 20 U/ml SUPERase·In (Invitrogen #AM2696), 1:200 Millipore protease inhibitor IV #539136). For the hairpin experiments in unstressed cells, samples were resuspended in polysome lysis buffer lacking heparin. For EDTA experiments, samples were resuspended in polysome lysis buffer lacking heparin and cycloheximide with 40mM EDTA to chelate Mg^2+^ and disrupt ribosomal complexes. The lysate was clarified by centrifugation at 3000 g for 30 s, and the clarified lysate was transferred to a new tube and aliquots were flash frozen in LN2.

A 10–50% continuous sucrose gradient in polysome gradient buffer (5 mM HEPES-KOH (pH 7.4), 140 mM KCl, 5 mM MgCl2, 100 μg/ml cycloheximide, 10 U/ml SUPERase·In, 0.5 mM TCEP) was prepared in SW 28.1 tubes (Seton #7042) using a Biocomp Gradient Master and allowed to cool to 4°C. Clarified lysate (200 μL) was loaded on top of the gradient, and gradients were spun in a SW28.1 rotor at 28,000 rpm for 3.5 hr at 4°C. Gradients were fractionated into 0.6mL fractions using a Biocomp Piston Gradient Fractionator with UV monitoring at 254 nm, and fractions were flash frozen in LN2. UV traces were normalized to the total signal starting with the 40S peak.

The samples were generated by pooling 50 μL of each fraction from the free fraction (before the monosome peak) and either separately pooling the fractions with 3+ ribosomes bound and the mono/di-some fractions (for the heat shock experiments), or by combining all ribosome-bound fractions together (azide and ethanol stresses). For the hairpin experiments in unstressed cells, samples were generated by pooling 75 μL from each pair of adjacent gradient fractions. The spike-in (50 ng of S. pombe total RNA) was then added to each pooled sample. RNA was purified via ethanol precipitation (final concentrations of 0.3 M sodium acetate pH 5.2, 0.3 μg/mL glycoblue (Invitrogen #AM9516), and 70% ethanol) at −20°C overnight followed by centrifugation at 4°C for 30 minutes at 21,000 g. The pellet was washed with 1 mL of 70% ethanol before being resuspended in water. The purified RNA was then treated with Dnase I (NEB) before purifying again using an NEB RNA clean and concentrate kit (NEB #T2030).

#### Membrane flotation assay

Assay was performed with some modifications based on previous work.^61^ ~100 mg of frozen yeast were transferred to a pre-chilled 2 ml Eppendorf “Safe-Lok” tube and lysed with a pre-chilled 7 mM stainless steel ball (Retsch #05.368.0035) by 5x90sx30Hz pulses in a Retsch MM100 mixer mill. Cells were chilled in liquid nitrogen (LN2) between pulses. Samples were resuspended in 10:1 (v/w) lysis buffer (20 mM HEPES-KOH (pH 7.4), 140 mM KCl, 5 mM MgCl2, 100 μg/ml cycloheximide, 10 U/ml SUPERase·In, 0.5 mM TCEP, 1% triton X-100). Lysis buffer was made lacking 1% triton X-100 when indicated. Lysate was clarified by centrifugation at 3,000g for 30 s and 250 μL of supernatant was mixed with 500 μL of 60% Optiprep iodixanol (Axis-shield). From this mixture, 600 μL were collected and dispensed at the bottom of SW 55 Ti tubes (Beckman Coulter #349622). Each tube was filled with 1.4 mL of 30% Optiprep with 100 μL of lysis buffer loaded on top. Samples were spun in SW 55 Ti rotor at 55,000 rpm for 2.5 hr at 4°C. Following centrifugation, gradients were manually fractioned starting from the top into 6 fractions of 350 μL. For each fraction, 50 μL was boiled in 2x Laemmeli buffer and 150 μL had a spike-in (50 ng of S. pombe total RNA) added prior to RNA purification via ethanol precipitation (final concentrations of 0.3 M sodium acetate pH 5.2, 0.3 μg/mL glycoblue (Invitrogen #AM9516), and 70% ethanol) at −20°C overnight followed by centrifugation at 4°C for 30 minutes at 21,000 g. The resulting pellet was resuspended in water and treated with DNase I (NEB) before being purified using an NEB RNA clean and concentrate kit (NEB #T2030).

#### Sucrose cushion ribosome occupancy analysis

The ribosome occupancy (fraction of mRNA bound to ribosome) for the induction reporters was measured by spinning lysate through a sucrose cushion. Around 100 mg of frozen yeast was transferred to a pre-chilled 2 ml Eppendorf “Safe-Lok” tube. Cells were lysed with a pre-chilled 7 mM stainless steel ball (Retsch #05.368.0035) by 5 x 90s x 30Hz pulses in a Retsch MM100 mixer mill, chilling in liquid nitrogen (LN2) between pulses. Sample was resuspended in 10:1 (v/w) polysome lysis buffer (20 mM HEPES-KOH (pH 7.4), 100 mM KCl, 5 mM MgCl2, 200 μg/mL heparin (Sigma #H3149), 1% triton X-100, 0.5 mM TCEP (Goldbio #TCEP25), 100 μg/mL cycloheximide (Sigma #C7698-5G), 20 U/ml SUPERase·In (Invitrogen #AM2696), 1:200 Millipore protease inhibitor IV #539136). The lysate was clarified by centrifugation at 3000 g for 30 s, and 500 μL clarified lysate was transferred to a new tube.

At this point the sample was split into +/− EDTA samples. For the +EDTA samples, 6 μL of 0.5 M EDTA (pH 8 in water) was added to 150 μL of clarified lysate and incubated on ice for 10 minutes. Then 100 μL of both samples (+/− EDTA) was gently added on top of 900 μL of matching sucrose cushion (5 mM HEPES-KOH (pH 7.4), 140 mM KCl, 5 mM MgCl2, 100 μg/ml cycloheximide, 10 U/ml SUPERase·In, 0.5 mM TCEP, 20% sucrose w/v, +/− 20 mM EDTA) and centrifuged for 60 minutes at 100,000 g in a TLA55 rotor (Beckman-Coulter) at 4°C. The top 250 μL of supernatant was removed as the supernatant sample and 100 μL of this was mixed with 300 μL Trizol LS. The remaining supernatant was discarded before resuspending the pellet in 100 μL water + 300 μL Trizol LS (pellet is 10x relative to supernatant). 1 ng of spike-in RNA was added to the pellet, but only 0.1 ng was added to the supernatant.

RNA was purified from the supernatant and pellet samples using Zymo Direct-Zol kits, then the abundances of target RNAs were quantified via qPCR as above. Ribosome occupancies were calculated by calculating the percentage of each transcript in the pellet, after correcting for the pelleting observed in the presence of EDTA (this separates EDTA-sensitive polysomes in the pellet from EDTA-insensitive condensates).

#### RNA sequencing

In general, DNase I treated RNA was prepared for sequencing using rRNA depletion (Illumina RiboZero (Illumina #MRZY1306) or Qiagen FastSelect (Qiagen #334215) followed by NEB NEBNext Ultra II (NEB #E7760) or Illumina TruSeq library preparation and Illumina platform sequencing. Specific methods for library preparation, sequencing and initial data analysis are described below and the method used for each sample is indicated in Table S2.

#### Sequencing analysis

##### Genome references

Saccharomyces cerevisiae reference genome files (S288C_reference_genome_R64-3-1_20210421) were downloaded from the Saccharomyces Genome Database.^89^ Schizosaccharomyces pombe reference genome files were downloaded from PomBase.^90^ When appropriate (see Table S2), the sequences of the NanoLuc spike-in or the mCherry and Clover reporters were included in the genome and transcriptome files for mapping.

##### Group A

See Table S2. Sequencing libraries were prepared by the University of Chicago Genomics Facility from DNase I treated RNA using Illumina RiboZero (Illumina #MRZY1306) and Illumina TruSeq library prep kits. Single end 50 bp sequencing was performed on an Illumina HiSeq 4000 sequencer.

Sequencing reads were trimmed using TrimGalore (v0.6.10, https://github.com/FelixKrueger/TrimGalore) using default settings (e.g. trim_galore –gzip –fastqc_args ’–outdir fastqc/’ -j 4 -o trimmed –basename FW32 EW_FW32_R1.fastq.gz). They were mapped using STAR v2.7.10b^82^ (e.g. STAR –outSAMtype BAM Unsorted –readFilesCommand gunzip -c –sjdbGTFfile saccharomyces_cerevisiae_R64-3-1_20210421_nofasta_geneid.gff –sjdbGTFtagExonParentTranscript Parent –sjdbGTFfeatureExon CDS –sjdbGTFtagExonParentGene gene_id –runThreadN 4 –alignMatesGapMax 20000 –limitBAMsortRAM1445804817 –genomeDir STAR_saccharomyces_cerevisiae_R64-3-1_20210421_allchrom –outFileNamePrefix mapped_reads/FW32/FW32_ –readFilesIn trimmed/FW32_trimmed.fq.gz). To generate estimated counts and transcript per million (TPM) values, sequencing reads were mapped to the yeast transcriptome using kallisto v0.48.0^83^ (e.g. kallisto quant -i Scerevisiae_orf_coding_all_Scerevisiae_rna_coding.fasta.idx -o kallisto_quant/FW32 –single -l 200 -s 1 –rf-stranded –bootstrap-samples=50 -t 1 trimmed/FW32_trimmed.fq.gz).

##### Group B

See Table S2. Sequencing libraries were prepared by from DNase I treated RNA using Qiagen FastSelect (Qiagen #334215), NEBNext Multiplex Oligos (UMI Adaptor RNA Set 1, NEB #E7335L) and NEBnext Ultra II Directional RNA library prep kits (NEB #E7760L). Paired end 200 bp sequencing with additional reads for dual 8/8 indices plus the 11nt UMI after the i7 index was performed on an Illumina NovaSeq 6000 at the University of Chicago Genomics Facility.

The unique molecular indices (UMIs) were extracted from fastq R2 using Umi-Tools v1.1.4^84^ and stored in fastq R1 and R3 (e.g. umi_tools extract –bc-pattern=XXXXXXXXNNNNNNNNNNN -I AD-JB-1S-HG02_S2_R2_001.fastq.gz –read2-in=AD-JB-1S-HG02_S2_R1_001.fastq.gz –read2-out=labeled_fastq/HG002/HG002_R1.umi.fastq. Sequencing reads were then trimmed using TrimGalore (v0.6.10, https://github.com/FelixKrueger/TrimGalore) using default settings (e.g. trim_galore –paired –gzip –fastqc_args ‘–outdir fastqc/’ -j 4 -o trimmed –basename HG002 labeled_fastq/HG002/HG002_R1.umi.fastq labeled_fastq/HG002/HG002_R3.umi.fastq). They were mapped using STAR v2.7.10b^82^ (e.g. STAR –outSAMtype BAM Unsorted –readFilesCommand gunzip -c –sjdbGTFfile spike_saccharomyces_cerevisiae_R64-3-1_20210421_geneid.gff3 –sjdbGTFtagExonParentTranscript Parent –sjdbGTFfeatureExon CDS –sjdbGTFtagExonParentGene gene_id –runThreadN 4 –alignMatesGapMax 20000 –limitBAMsortRAM 1445804817 –genomeDir STAR_spike_saccharomyces_cerevisiae_R64-3-1_20210421 –outFileNamePrefix mapped_reads/HG002/HG002_ –readFilesIn trimmed/HG002_val_1.fq.gz trimmed/HG002_val_2.fq.gz). Umi-Tools was then used again to deduplicate the reads (e.g. umi_tools dedup –stdin=mapped_reads/HG002/HG002_Aligned_Sorted.out.bam –chimeric-pairs=discard –unpaired-reads=discard –spliced-is-unique –paired -S mapped_reads/HG002/HG002_Aligned.sortedByCoord.dedu-p.out.bam). The reads were split again into fastq files using samtools v1.16.1,^85^ and then estimated counts and TPMs were generated using kallisto v0.48.0^83^ (e.g. kallisto quant -i spike_Scerevisiae_orf_coding_all_Scerevisiae_rna_coding.fasta.idx -o kallisto_quant/HG002 –rf-stranded –bootstrap-samples=50 -t 1 mapped_reads/HG002/HG002_Aligned_dedup_R1.fastq.gz mapped_reads/HG002/HG002_Aligned_dedup_R3.fastq.gz).

##### Group C

See Table S2. Sequencing libraries were prepared by the University of Chicago Genomics Facility from DNase I treated RNA using Qiagen FastSelect (Qiagen #334215) and Illumina Stranded mRNA Prep (Illumina #20020595) kits. Paired end 200 bp sequencing was performed on an Illumina NovaSeq 6000.

Sequencing reads were trimmed using TrimGalore (v0.6.10, https://github.com/FelixKrueger/TrimGalore) using default settings (e.g. trim_galore –paired –fastqc_args ’–outdir fastqc/’ -j 4 -o trimmed –basename F02 AD-JB-F02_S44_R1_001.fastq.gz AD-JB-F02_S44_R2_001.fastq.gz). They were mapped using STAR v2.7.10b^82^ (e.g. STAR –outSAMtype BAM Unsorted –readFiles-Command gunzip -c –sjdbGTFfile spike_saccharomyces_cerevisiae_R64-3-1_20210421_geneid.gff3 –sjdbGTFtagExon-ParentTranscript Parent –sjdbGTFfeatureExon CDS –sjdbGTFtagExonParentGene gene_id –runThreadN 4 –alignMatesGapMax 20000 –limitBAMsortRAM 1445804817 –genomeDir STAR_spike_saccharomyces_cerevisiae_R64-3-1_20210421 –outFileName-Prefix mapped_reads/F02/F02_ –readFilesIn trimmed/F02_val_1.fq.gz trimmed/F02_val_2.fq.gz). The estimated counts and TPMs were generated using kallisto v0.48.0^83^ (e.g. kallisto quant -i spike_Scerevisiae_orf_coding_all_Scerevisiae_rna_coding.fasta.idx -o kallisto_quant/F02 –fr-stranded –bootstrap-samples=50 -t 1 trimmed/F02_val_1.fq.gz trimmed/F02_val_2.fq.gz).

#### Calculation of pSup

Public code for calculating pSup from sequencing data is available here: https://github.com/jabard89/sedseqquant. The statistical model used to estimate the proportion in supernatant (pSup) was based on that used in Wallace et al.^34^ For each fractionated sample, the number of counts of mRNA within each fraction—total (T), supernatant (S), and pellet (P)—were extracted from RNA-sequencing data (see “sequencing analysis” section above). While mRNAs are expected to obey conservation of mass in the original fractionated lysate (*T_i_* = *S_i_* + *P_i_* for mRNA species i), this assumption does not hold in the ratios of abundances directly inferred from the data. Instead, for a particular experiment, *T_i_* = *a_S_S_i_* + *a_P_P_i_* where we refer to the per-experiment constants *a_S_* and *a_P_* as mixing ratios which reflect differential processing and measurement of individual fractions. In order to estimate mixing ratios, and thus recover the original stoichiometry, we assume conservation of mass for each mRNA in the sample, and then estimate the mixing ratios under this constraint using a Bayesian model.^91^ We assume negative binomial (NB) noise for each count measurement, and log-normal underlying distribution of mRNA abundance. Specifically, we model counts as follows:

$$log\left(T_{i}\right)∼NB\left(log\left(a_{S}S_{i}+a_{P}P_{i}\right),⊘\right)$$

where

*T_i_* = measured abundance of mRNA *i*,

*S_i_* = measured abundance in supernatant of mRNA *i*,

*P_i_* = measured abundance in pellet of mRNA *i*,

*a_S_* = mixing ratio of supernatant sample,

*a_P_* = mixing ratio of pellet sample

With the following priors:

$$a_{S}∼\tau (1,1)$$

$$a_{P}∼\tau (1,1)$$

⊘∼Cauchy(0,3)

We implemented the model above in R using the probabilistic programming language STAN, accessed using the rstan package^92,93^ and used all mRNA with *counts* > 20 to estimate mixing ratios for each sample. These mixing ratios were then used to calculate the pSup for mRNA *i*:

$$p\;Sup_{i}=\frac{a_{S}S_{i}}{a_{S}S_{i}+a_{P}P_{i}}$$

#### Differential sedimentation and escape scores

To calculate the differential sedimentation (ΔSed) and escape (eSed) scores, which capture a stress-dependent difference from the treatment, we first calculate a windowed mean over transcript length of log-odds pSup in the mock (untreated) condition. Each window is 0.02 of the full range of transcript lengths on a log scale.

For a transcript with length L, we take the mean of the log-odds pSup values for all transcripts within a window centered on log L; these means μ(L,T) are calculated for all values of L in the transcriptome. The log-odds scale, log p/(1–p) if pSup = p, is used to map proportions on the unit interval to the real line, preventing compression at high or low pSup values. We then compute the standard deviation σ of all transcript pSup values from the windowed mean for the corresponding length.

Given the resulting quantities:

lopSup(x,T) = log-odds pSup [logp/(1−p)if pSup=p] of gene x after treatment T

μ(L,T) = mean lopSup in window around length L after treatment T,

σ(L,T) = standard deviation lopSup in window around length L after treatment T,

σ(T) = standard deviation of lopSup(x,T = control) – μ(L,control) over all genes x

the differential sedimentation ΔSed for gene x, with transcript length L, after treatment T is

ΔSed(x,T)=[lopSup(x,control)−lopSup(x,T)]/σ(control)

and the escape from sedimentation eSed for gene x after treatment T is

eSed(x,T)=[(lopSup(x,T)−μ(L,T))−(lopSup(x,control)−μ(L,control))]/σ(control)

Intuitively, ΔSed captures changes in sedimentation due to treatment in units of σ(control). ΔSed = 0 for the control condition. Escape score eSed captures the treatment-induced difference in sedimentation relative to transcripts of the same length; eSed > 0 indicates less sedimentation than the average (escape), and eSed < 0 indicates more sedimentation than the average.

#### Other bioinformatic analyses

##### Transcript features

Transcript features were extracted from Saccharomyces Genome Database.^89^ Targets of HSF1 and MSN2/4 were based off those reported in Pincus et al. 2018^44^ and Solís et al.^45^ Transcript UTR lengths were taken as the median value reported by long read transcript sequencing in Pelechano et al.,^94^ or, when no data was reported, the median UTR length in yeast was used as the default. Pombe transcript lengths, including the lengths of the UTRs, was taken from PomBase.^90^

##### Transcript abundance

The transcript abundance is reported as the geometric mean of the TPM value for two biological replicates, estimated by kallisto analysis of the Total fraction for each sample. Changes in transcript abundance were calculated using DESeq2.^86^

##### sedScore calculation

In order to calculate sedScores, the pSup for each transcript was converted to a log-odds scale, and transcripts were arranged by their length (including UTRs), and then binned into groups of 100. For each transcript in the bin, the standard deviation from the mean within the bin was used to calculate a Z-score. Individual Z-scores from two biological replicates were calculated and then averaged together for the final reported sedScore.

##### Ribosome occupancy

Because Polysome-seq data was collected with spike-in values for each fraction (Total, Free, Mono/Poly), it is possible to calculate the absolute ribosome occupancy (% of a transcript which is bound to at least one ribosome) for each transcript. This value is calculated by normalizing transcript abundance for each fraction (TPMs output by kallisto) to the median abundance of the spike-in transcripts. All S. pombe spike-in transcripts with more than 100 estimated counts were used to calculate the spike-in abundance. The ribosome occupancy is then calculated as ${abundance}_{\text{bound}}/({abundance}_{bound}+{abundance}_{\text{free}})$.

##### Ribosome association

In stressed samples, it is possible that condensed RNA pellets to the bottom of the sucrose gradient, making it difficult to calculate the absolute ribosome occupancy. Thus, for stressed samples, we calculate a “ribosome association” score which is ${TPM}_{\text{rib. bound}}/{TPM}_{Total}$.^95^ This metric is similar to “translation efficiency” scores calculated for ribosome profiling studies.^58^ The change in ribosome association upon stress was calculated using DESeq2,^86^ similar to reported methods for calculating changes in translation efficiency using DESeq2.^96^

##### RNA structure analysis

The sequence for the 5′ UTR + the first 20 nucleotides of the CDS was extracted using the 5′ UTR lengths described above from Pelechano et al.^94^ The folding energy for each UTR was then calculated using RNAFold from the ViennaRNA package.^87^ Because the folding energy correlates directly with length, a normalized structure score was calculated for each transcript by dividing the calculated folding free energy by the length of the UTR.

#### Inducible reporter genes

Reporters for pulsed induction were generated by Gibson assembly of gene fragments with a TET-inducible promoter designed for tight control of induction levels.^81^ Assembly pieces were derived either from gene fragments ordered from IDT or Twist Biosciences or from PCR amplification of other plasmids. Fragments were assembled into backbones generated by golden gate cloning using protocols and plasmids from the Yeast Toolkit,^97^ and the plasmids were sequenced by overlapped Sanger sequencing. Plasmids were linearized with NotI prior to transformation.

The *PMU1* reporter contains the 5′ UTR and 3′ UTR of the native *PMU1* gene and the CDS is a fusion of the *PMU1* CDS with nanoluciferase-PEST.^98^ The *HSP26* reporter contains the 5′ UTR and 3′ UTR of the native *HSP26* gene, but the CDS is a fusion of the *TPI1* CDS and nanoluciferase-PEST. The *TPI1* fusion was used to avoid potential artifacts caused by a large pre-induction of *HSP26* molecular chaperone and because *TPI1* is well translated during stress and of a similar length (645 nt for *HSP26* vs 745 nt for *TPI1*). Reporters were integrated at the HO locus using hygromycin selection in a strain of yeast containing a C-terminal auxin tag on Sui2, along with the inducible TIR1 ligase at the LEU locus, and the TetR protein at the his locus (see key resources table for full genotype).

For induction of reporters concurrently with stress, 1 μM anhydrotetracycline (aTC, Cayman #CAYM-10009542-500) was added from a 10 mM stock prepared in DMSO at the beginning of the stress. For pre-induced samples, 0.1 μM aTC was added to yeast in SCD at OD_600_ = 0.2 and samples were incubated at 30°C for 45 minutes. Samples were then either washed 3x with SCD via centrifugation, or 1x via vacuum filtration before resuspending in prewarmed SCD. Stress was then initiated 30 minutes after washing had begun to ensure complete shutoff of reporter transcription. Samples were then fractionated as described above either using the Sed-seq protocol to calculate pSup or the sucrose cushion fractionation to calculate ribosome occupancy.

#### Engineering solubility reporters

Solubility reporters were engineered using the Yeast Toolkit^97^ (see key resources table). Variable 5′ UTRs were engineered depending on the construct and genetically integrated in front of two copies of Clover, all driven by the constitutive TPI1 promoter and with the TPI1 3′ UTR. Each reporter construct also had a copy of mCherry with a TPI1 promoter, 5′ UTR and 3′ UTR. This construct was inserted into the Leu2 locus with leucine selection.

Steady state protein levels were measured using flow cytometry by normalizing the Clover signal to the mCherry signal in each cell. Data was analyzed with a custom script using FlowCytometryTools in python and then exported and plotted in R. The Sed-seq protocol was used to measure the condensation behavior of each strain. Steady state mRNA levels were extracted from the Total sample of the Sed-seq experiment and translation efficiency was calculated as the steady state protein level divided by steady state RNA level.

#### Auxin-mediated depletion strains

Auxin induced degron depletions were adapted from the approach in Mendoza-Ochoa et al.^51^ In short, the endogenous protein of interest was genetically engineered to contain the degron tag in a strain of yeast in which a β-estradiol inducible TIR1 ligase had been genetically integrated at the LEU locus. Some of the strains contained the original Oryza sativa TIR1 (OsTIR1), while others used a variant engineered for more specificity OsTIR1(F74G)^52^ as indicated in key resources table. The auxin-FLAG degrons were installed at either the 5’ or 3’ end of genes using CRISPR plasmids from the yeast toolkit. A PCR-generated DNA template was co-transformed with a Cas9 and gRNA containing URA3 selectable plasmid as previously described.^97,99^ The CRISPR integrations were verified by PCR and Sanger sequencing and the URA3 plasmid was removed by selecting for colonies which did not grow on URA plates.

For depletion experiments, yeast were grown at 30°C in YPD to OD_600_ = 0.1. To induce TIR1 ligase, 5 μM β-estradiol (10 mM stock in DMSO) or an equivalent volume of DMSO (for mock treatment) was added to each culture and they were incubated for 75 minutes. To induce degradation, auxin added was either 100 μM of Indole-3-acetic acid (IAA) sodium salt (Sigma #I5148, 250 mM stock in DMSO) or 5 μM of 5-Ph-IAA (Medchemexpress #HY-134653, 5 mM stock in DMSO). After 2 hours of auxin exposure, cells were temperature treated and then harvested and fractionated as normal.

#### Radiolabeling quantification of translation

Yeast cells were cultured overnight in YPD until they reached an OD600 = 0.1. Auxin-inducible yeast strains were then treated with beta-estradiol and auxin, as detailed above, then translation was measured following a published protocol.^100^ After a 1.5-hour depletion period, 1 mL of sample was transferred to 1.5mL tubes, then 1 μCi/mL of mixed 35S-L-methionine and 35S-L-cysteine media were added to each sample (Perkin-Elmer EasyTag #NEG772002MC). Samples were incubated for 30 minutes at 30°C with shaking (15 minutes for heat shocks), then cells were treated with 200 μL of 50% trichloroacetic acid (TCA), chilled on ice for 10 minutes, heated at 70°C for 20 minutes, and cooled again for 10 minutes. The samples were subsequently collected on glass microfiber filters (Sigma #WHA1823025) loaded onto a vacuum manifold (Millipore #XX2702550), washed with 3x 5 mL 5% TCA and 2x 5mL 95% ethanol, and air-dried for at least 12 hours at room temperature. Filters were then immersed in scintillation fluid (Perkin Elmer #6013179), and radioactivity levels were quantified in “counts per minute” through liquid scintillation counting on a Tri-Carb machine.

#### Western blotting

Western blots were performed as described in a published protocol.^101^ For each sample, 1mL of yeast culture was spun down at 2500 g for 2 minutes, and the pellet was resuspended in 50 μL of 100 mM NaOH. The samples were incubated for 5 minutes at RT, spun at 20,000g for 1min, and resuspended in 50 μL of 1x Laemmli buffer (Bio-rad #1610737) with 5% β-mercaptoethanol. Samples were then boiled for 3 minutes, clarified at 20,000 g for 2 minutes and 15 μL was loaded onto a 4-20% tris-glycine SDS-PAGE gel (Bio-rad #5671094). Proteins were then transferred to nitrocellulose (Sigma #10600001) using a wet transfer apparatus (Bio-rad #1704070). The membrane was blocked for 1 hour with 5% milk in TBST buffer, then incubated rocking overnight at 4°C with 1:3000 dilution of anti-FLAG antibody (Sigma #F1804) and 1:10,000 dilution of anti-PGK1 antibody (Invitrogen #459250) or a 1:3000 dilution of anti-GFP antibody (Fischer #A-11122) in 5% milk solution. Westerns were visualized using 1:20,000 dilutions of fluorophore conjugated secondaries (Licor #926-32212 and #925-68073) and visualized on a Licor Odyssey CLx. Band intensities were quantified in ImageJ and normalized to PGK1 signal. For Dpm1, primary incubation was done at 1:1000 for 48 hours.

#### Fluorescence microscopy and stress granule quantification

Standard confocal microscopy was completed as in Wallace et al.,^34^ generally using Pab1-Clover as the SG marker unless otherwise noted. Cells were grown to log-phase as previously described. 1mL of cells were transferred to 1.5mL Eppendorf tubes. For heat stress, cells were shocked in a heat block, spun down in a microfuge, and 950 uL of supernatant were removed. For azide stress, 10% (w/v) azide or water was added directly to the 1mL of cells as indicated. For ethanol stress, cells were spun down in microfuge and resuspended in media with appropriate amounts of ethanol. 1.5 uL of treated cells were then placed on a glass slide and imaged immediately. For AID treatment, cells were treated as described above, and were imaged immediately after a 2 hour exposure to Auxin. For cycloheximide treatment, cells were exposed to 100 ug/mL of cycloheximide for 10 minutes, stressed for 10 minutes, and then imaged immediately. Cells were imaged on an Olympus DSU spinning disc confocal microscope using a 100x 1.45 TIFM oil objective (PlanApo) and the FITC filter cube for the Clover fluorophore in Z-stacks. Representative images are maximum projections of the collected z-stacks. Maximum projection images of the cells were used to quantify the number of stress granules per cell using CellProfiler.

Because automated counting scored some unstressed (30°C) cells as having multiple SGs, and all conditions show some degree of cell-to-cell variability, we scored populations of cells as SG-negative if the median number of SGs per cell was zero, and as SG-positive otherwise. Using this threshold, unstressed cells are SG-negative and cells shocked at 46°C are SG-positive (Figure 7A).

#### Single-molecule fluorescence in situ hybridization (smFISH)

Custom Stellaris^®^ RNA FISH Probes were designed against SSB1, SSA4, HSP104, and ADD66 by utilizing the Stellaris^®^ RNA FISH Probe Designer (Biosearch Technologies, Inc., Petaluma, CA) available online at www.biosearchtech.com/stellarisdesigner (Table S1). Each Stellaris FISH Probe set was labeled with Quasar670 (Biosearch Technologies, Inc.). smFISH was done as previously described.^102,103^ Yeast cultures were grown to an OD of 0.3-0.4 in SCD, spun down at 3k g for 3 min. Cells were then suspended into 4mL of culture and Oregon Green HaloTag reagent (Promega #G2801) was added to a final concentration of 2uM. Cells were then resuspended and split into final cultures of 25 mL. Cells were then spun again at 3000g for 3min, and 23mL were removed, such that 2mL of media remained. Cells were then stressed as stated before. 19.85mL of pre-warmed media was then added to each falcon tube, and 3.15 mL of 4% paraformaldehyde (Electron Microscopy Sciences #15714) was immediately added. Cells were incubated at room temperature for 45 min at room temperature, gently rocking. Cells were spun down at 4°C and washed with ice-cold buffer B. Cells were resuspended into 1mL of Buffer B (1.2M sorbitol, 100mM KHPO4, pH = 7.5) then transferred to a 12-well plate. Cells were additionally crosslinked in a Spectrolinker UV Crosslinker at a wavelength of 254nm by exposure to 100 mJ/cm^^2^ twice with 1 min break in between.^104^ Cells were pelleted for 3min at 2000rpm and then resuspended into spheroplast buffer (1.2 M sorbitol, 100 mM KHPO4, pH = 7.5, 20mM ribonucleoside-vanadyl complex (NEB # S1402S), 20mM B-mercaptoethanol). 25U/OD of lyticase (Sigma #L2524-10KU) were added to each sample. Cell digestion was performed at 30°C and was monitored using a benchtop phase contrast microscope, such that cells were about 50%-70% digested. Digestion was stopped by spinning cells at 4°C for 3min at 2000 rpm and two washes twice in ice cold buffer B and resuspended in 1mL Buffer B. 250 uL of cells were placed onto a poly-L lysine coated coverslip and incubated at 4C for 1hr. Cells were washed with 2mL of Buffer B and then stored in ice-cold 70% ethanol for at least 3 hours. Coverslips were rehydrated in 2xSSC and then washed twice in pre-hybridization buffer (2x SSC + 5% formamide (Sigma #344206-100ML-M)) for 5 minutes each. A mixture of 0.125uL of 25uM smFISH probes, and 2.5uL of 10mg/ml yeast tRNA (Thermo #AM7119) and 2.5uL of 10mg/mL salmon sperm DNA (Thermo #15632011) was dehydrated in a Speedvac at 45°C. The dried pellet was rehydrated was resuspended in 25 μl hybridization mix (10% formamide, 2×SSC, 1mg/mL BSA, 10 mM Ribonucleoside–vanadyl complex (NEB # S1402S) and 5 mM NaHPO4, pH 7.5) and boiled at 95 °C for 2 min. 18uL of resuspended probes were spotted onto a piece of Parafilm and coverslips were placed cell-side down into hybridization mixture. Hybridization occurred at 37°C for 3 hours. Coverslips were then washed at 37°C for 15min in 2x SSC + 5% formamide, then in 2x SSC buffer, then 1xSSC buffer. They were then submerged in 100% Ethanol, dried, and then mounted into ProLong Gold antifade with DAPI (Thermo P36941).

#### smFISH image acquisition and analysis

smFISH images were taken on a Nikon TiE microscope with a CFI HP TIRF objective (100x, NA 1.49, Nikon), and an EMCCD (Andor, iXon Ultra 888). Nikon TiE epifluorescent microscope. Samples were excited using the 647nm laser (Cobolt MLD) (~15-20 mW for 200-300ms), poly-A FISH was imaged using the 561nm laser (Coherent Obis) (~15-20 mW for 200-300ms), and Pab1-Halotag signal was imaged with a 488nm laser (Cobolt MLD) (~10-15 mW for 200-300 ms), and DAPI (CL2000, Crystal Laser) (~5-10 mW for 100 ms). Imaging of the nucleus was done using the 405nm laser and DIC images were taken as well. Z-stacks of 21 planes, 2uM thick were obtained. Images were analyzed using FISH-quant.^105^ Briefly, RNA spots were identified using Big-FISH.^105^ For the smFISH colocalization analysis, RNA spot intensities were normalized by dividing by the mean intensity of each cell. For each RNA spot, the mean Pab1 intensity in a 3x3 pixel square around the centroid was calculated. The Pab1 intensity was then measured for 100 random locations in the cell in 3x3 pixel locations. Finally, a distribution was calculated for both the random Pab1 signal and the Pab1 signal that corresponds to a RNA spot. The Z-score of the mean intensity of the Pab1 signal in a RNA spot compared to the Pab1 signal in a random spot was compared, and this is termed the ‘colocalization score’. Each Z-score is calculated independently for each cell to account for different background intensities, and the average shown is for every cell. In Figure 2, the colocalization score is plotted as the mean of all cells in each condition. Pairwise Welch’s t-tests were performed, and the Holm method was used to adjust p-values to correct for multiple comparisons. Significance thresholds were defined as follows: N.S. (p ≥ 0.05); * (p < 0.05); ** (p < 0.01); *** (p < 0.001).

#### Fitting of mRNA and mRNP condensation

The underlying biophysical model for pSup in the absence of condensation is $pSup(g)=1-\beta L_{g}x$ for a mRNA transcript encoded by gene g, of length $L_{g}$. In conditions where there is mRNA condensation, governed by parameter μ per-transcript and ν per-nucleotide, the model is: $pSup(g)=\left(1-\beta L_{g}x\right)e^{-\left(\mu +vL_{g}\right)}$. These models were fitted to sedimentation on the log-odds(pSup) scale, i.e. approximating the log-odds sedScore as normally distributed. Non-linear least squares fits were performed using the nls function in R. See supplemental text for details.

#### Statistical analyses

Unless otherwise stated, all experiments were performed as at least two biological replicates, and the mean or geometric mean value (for log-distributed transcript abundance data) was calculated from the replicates. Unless otherwise noted, all correlation values are reported as Spearman’s rank correlation coefficient and significance tests comparing groups of data points were performed using a Wilcoxon rank-sum test, with a Bonferroni correction when multiple groups were being compared (*P < 0.05, **P < 0.01, ***P < 0.001. ’N.S.’ denotes not significant (P ≥ 0.05).

### QUANTIFICATION AND STATISTICAL ANALYSIS

Unless otherwise stated, all experiments were performed as at least two biological replicates, and the mean or geometric mean value (for log-distributed transcript abundance data) was calculated from the replicates. Unless otherwise noted, all correlation values are reported as Spearman’s rank correlation coefficient and significance tests comparing groups of data points were performed using a Wilcoxon rank-sum test, with a Bonferroni correction when multiple groups were being compared (*P < 0.05, **P < 0.01, ***P < 0.001. ’N.S.’ denotes not significant (P ≥ 0.05).

## Supplementary Material

1

2

3

Supplemental information can be found online at https://doi.org/10.1016/j.molcel.2025.11.003.

## Figures and Tables

**Table:** KEY RESOURCES TABLE

REAGENT or RESOURCE	SOURCE	IDENTIFIER
Antibodies		
Mouse monoclonal Anti-FLAG M2	Sigma-Aldrich	Cat#F1804; RRID: AB_262044
Mouse monoclonal Anti-PGK1	Invitrogen	Cat#459250; RRID: AB_2532235
Rabbit monoclonal Anti-GFP	Fisher	Cat#A-11122; RRID: AB_2215699
Donkey Anti-mouse IgG secondary, IRDYE 800CW	Licor	Cat#926-32212; RRID: AB_2716622
Donkey Anti-rabbit IgG secondary, IRDYE 680RD	Licor	Cat#925-68073; RRID: AB_2716687
Chemicals, peptides, and recombinant proteins		
Cycloheximide	Sigma-Aldrich	Cat#C7698-5G
Sodium azide	Fisher	Cat#BP922I
Trizol LS	Fisher	Cat#10296028
Heparin	Sigma-Aldrich	Cat#H3149
Tris(2-carboxyethyl) phosphine (TCEP)	GoldBio	Cat#TCEP25
SUPERase·In	Invitrogen	Cat#AM2696
Protease Inhibitor IV	Millipore	Cat#539136
60% (w/v) OptiPrep Iodixanol	Axis-Shield	Cat#1114542
Anhydrotetracycline	Cayman	Cat#CAYM-10009542-500
Beta-estradiol	Santa Cruz	Cat#sc-204431A
Indole-3-acetic acid sodium salt	Sigma-Aldrich	Cat##I5148
5-Ph-IAA	Medchemexpress	Cat#HY-134653
EasyTag EXPRESS35S Protein Labeling Mix, [35S]	Perkin-Elmer	Cat#NEG772002MC
Scintillation fluid Ultima Gold F	Perkin-Elmer	Cat#6013179
Laemmli buffer	Bio-Rad	Cat#1610737
HaloTag Oregon Green Ligand	Promega	Cat#G2801
Paraformaldehyde 32% Aqueous Solution	Electron Microscopy Sciences	Cat#15714
Ribonucleoside Vanadyl Complex	New England Biolabs	Cat#S1402S
Lyticase	Sigma-Aldrich	Cat#L2524-10KU
Formamide	Sigma-Aldrich	Cat#344206-100ML-M
Critical commercial assays		
Ribo-Zero Gold rRNA (Yeast)	Illumina	Cat#MRZY1306
QIAseq FastSelect -rRNA Yeast Kit	Qiagen	Cat#334215
NEBNext^®^ Ultra^™^ II Directional RNA Library Prep Kit for Illumina	New England Biolabs	Cat#E7760
TruSeq RNA Sample Prep Kit	Illumina	Cat#FC-122-1001
NEBNext Multiplex Oligos; UMI Adaptor RNA Set 1	New England Biolabs	Cat#E7335L
iScript^™^ Select cDNA Synthesis Kit	Bio-Rad	Cat#1708897
LunaScript RT Supermix Kit	New England Biolabs	Cat#E3010L
Primetime gene expression master mix	IDT	Cat#1055771
Frozen-EZ Yeast Transformation II Kit	Zymo Research	Cat#T2001
Q5 Polymerase	New England Biolabs	Cat#M0494S
T7 Highscribe Kit	New England Biolabs	Cat#E2040S
Deposited data		
Raw sequencing data	This manuscript	GEO: GSE265963
Raw imaging data	This manuscript	doi: 10.17632/hby6drmgy9.1
Analysis and figure making scripts	This manuscript	https://github.com/drummondlab/RNACondensation2025/
Experimental models: Organisms/strains		
BY4741; MATa ura3Δ0 leu2Δ0 his3Δ1 met15Δ0	Brachmann et al.^78^	BY4741
BY4742; MATα ura3Δ0 leu2Δ0 his3Δ1 lys2Δ0	Brachmann et al.^78^	BY4742
Pab1-HaloTag; MATa ura3Δ0 leu2Δ0 his3Δ1 met15Δ0 pab1::PAB1-HaloTag	This manuscript	yJB001
Pab1-Clover; MATα ura3Δ0 leu2Δ0 his3Δ1 lys2Δ0 pab1::PAB1-Clover pTEF-KanMX	Wallace et al.^34^	yAER020
PRT1-AID*-3xflag+pZTRL; MATɑ ura3Δ0 leu2::pZ4EV-OsTIR1_Z4EV-ATF_LEU2 his3Δ1 lys2Δ0 PRT1::PRT1-AID*-3xflag	This manuscript	yJB42 (eIF3b)
AID*-TIF3-3xflag+pZTRL; MATɑ ura3Δ0 leu2::pZ4EV-OsTIR1_Z4EV-ATF_LEU2 his3Δ1 lys2Δ0 TIF3::TIF3-AID*-3xflag	This manuscript	yHG55 (eIF4b)
CDC33-AID*-3xflag+pZTRL; MATɑ ura3Δ0 leu2::pZ4EV-OsTIR1_Z4EV-ATF_LEU2 his3Δ1 lys2Δ0 CDC33::CDC33-AID*-3xflag	This manuscript	yHG38 (eIF4E)
TIF1-AID*-3xflag+pZTRL; MATɑ ura3Δ0 leu2::pZ4EV-OsTIR1_Z4EV-ATF_LEU2 his3Δ1 lys2Δ0 TIF1::TIF1-AID*-3xflag tif2Δ::KANMX	This manuscript	yHG72 (eIF4A)
SUI2-AID-3xFlag + pJB773; MATɑ ura3Δ0 leu2::pZ4EV-OsTIR1F74G_Z4EV-ATF_LEU2 his3Δ1 lys2Δ0 SUI2::SUI2-AID*-3xflag	This manuscript	yJB143 (eIF2α)
TIF5-AID-3xFlag + pJB773; MATɑ ura3Δ0 leu2::pZ4EV-OsTIR1F74G_Z4EV-ATF_LEU2 his3Δ1 lys2Δ0 TIF5::TIF5-AID*-3xflag	This manuscript	yJB147 (eIF5)
AID*-FUN12-3xFlag + pJB773; MATɑ ura3Δ0 leu2::pZ4EV-OsTIR1F74G_Z4EV-ATF_LEU2 his3Δ1 lys2Δ0 FUN12::AID-FUN12*-3xflag	This manuscript	yJB148 (eIF5B)
TIF4631-AID*-3xFLAG+pJB773; MATɑ ura3Δ0 leu2::pZ4EV-OsTIR1F74G_Z4EV-ATF_LEU2 his3Δ1 lys2Δ0 TIF4631::TIF4631-AID*-3xFLAG tif4632Δ::KANMX	This manuscript	yJB262 (eIF4G)
Pbp1-GFP; MATa his3Δ1 leu2Δ0 met15Δ0 ura3Δ0 pbp1:: Pbp1-GFP	Huh et al.^79^	Pbp1-GFP
yJB42+Pab1-Clover; MATɑ ura3Δ0 leu2::pZ4EV-OsTIR1_Z4EV-ATF_LEU2 his3Δ1 lys2Δ0 PRT1::PRT1-AID*-3xflag pab1::PAB1-Clover	This manuscript	yJB265
Weakest hairpin; MATa ura3Δ0 his3Δ1 met15Δ0 leu2::LEU2_5′weakest hairpin-cds2xClover-3′TPI1_5′TPI1-cdsmCherry-3′TPI1	This manuscript	yHG005
Weak hairpin; MATa ura3Δ0 his3Δ1 met15Δ0 leu2::LEU2_5′weak hairpin-cds2xClover-3′TPI1_5′TPI1-cdsmCherry-3′TPI1	This manuscript	yHG006
Medium harpin; MATa ura3Δ0 his3Δ1 met15Δ0 leu2::LEU2_5′ medium hairpin-cds2xClover-3′ TPI1_5′ TPI1-cdsmCherry-3′ TPI1	This manuscript	yHG007
Strongest hairpin; MATa ura3Δ0 his3Δ1 met15Δ0 leu2::LEU2_5′ strongest hairpin-cds2xClover-3′ TPI1_5′ TPI1-cdsmCherry-3′ TPI1	This manuscript	yHG008
No hairpin; MATa ura3Δ0 his3Δ1 met15Δ0 leu2::LEU2_5′ no hairpin-cds2xClover-3′ TPI1_5′ TPI1-cdsmCherry-3′ TPI1	This manuscript	yHG010
GCN4 reporter; MATa ura3Δ0 his3Δ1 met15Δ0 leu2::LEU2_5′ GCN4-cds2xClover-3′ TPI1_5′ TPI1-cdsmCherry-3′ TPI1	This manuscript	yHG026
GCN4 5xmut; MATa ura3Δ0 his3Δ1 met15Δ0 leu2::LEU2_5′ GCN4(5xmut)-cds2xClover-3′ TPI1_5′ TPI1-cdsmCherry-3′ TPI1	This manuscript	yHG027
Tet inducible PMU1 reporter; MATɑ ura3Δ0 leu2::pZ4EV-OsTIR1_Z4EV-ATF_LEU2 his3::TetR,TetR-Tup1,HIS3MX lys2Δ0 SUI2::SUI2-AID*-3xflag ho::pTETO-5′ PMU1-cdsPMU1-nanoluc-3′ PMU1,TPI-firefly,hygR	This manuscript	yJB236
Tet inducible HSP26 reporter; MATɑ ura3Δ0 leu2::pZ4EV-OsTIR1_Z4EV-ATF_LEU2 his3::TetR,TetR-Tup1,HIS3MX lys2Δ0 SUI2::SUI2-AID*-3xflag ho::pTETO-5′ HSP26-cdsTPI1-nanoluc-3′ HSP26,TPI-firefly,hygR	This manuscript	yJB240
Oligonucleotides		
See Table S1 for sequences	Biosearch Technologies	smFISH SSA4
See Table S1 for sequences	Biosearch Technologies	smFISH SSB1
See Table S1 for sequences	Biosearch Technologies	smFISH HSP104
See Table S1 for sequences	Biosearch Technologies	smFISH ADD66
See Table S3 for sequences	IDT	qPCR PGK1
See Table S3 for sequences	IDT	qPCR BEM2
See Table S3 for sequences	IDT	qPCR HAC1
See Table S3 for sequences	IDT	qPCR GCN4
See Table S3 for sequences	IDT	qPCR HSP26
See Table S3 for sequences	IDT	qPCR Nanoluciferase spike In
See Table S3 for sequences	IDT	qPCR yo-Nanoluciferase reporter
See Table S3 for sequences	IDT	qPCR Clover
Recombinant DNA		
Yeast plasmid; Cas9 editing	Vyas et al.^80^	pV1382
Yeast plasmid; Inducible OsTIR1	Mendoza-Ochoa et al.^51^	pZTRL
Yeast plasmid; OsTIR1F74G leu2 int leu	This manuscript	pJB773
Yeast plasmid; TETO system	Azizoglu et al.^81^	FRP2371
Yeast plasmid; Weakest hairpin	This manuscript	pyHG005
Yeast plasmid; Weak hairpin	This manuscript	pyHG006
Yeast plasmid; Medium harpin	This manuscript	pyHG007
Yeast plasmid; Strongest hairpin	This manuscript	pyHG008
Yeast plasmid; No hairpin	This manuscript	pyHG010
Yeast plasmid; GCN4 reporter	This manuscript	pyHG026
Yeast plasmid; GCN4 5xmut	This manuscript	pyHG027
Yeast plasmid; Tet inducible PMU1 reporter	This manuscript	pJB801
Yeast plasmid; Tet inducible HSP26 reporter	This manuscript	pJB805
Software and algorithms		
TrimGalore (v0.6.10)	The Babraham Institute	https://github.com/FelixKrueger/TrimGalore
STAR v2.7.10b	Dobin et al.^82^	https://github.com/alexdobin/STAR
Kallisto v0.48.0	Bray et al.^83^	https://github.com/pachterlab/kallisto
Umi-Tools v1.1.4	Smith et al.^84^	https://github.com/CGATOxford/UMI-tools
SAMtools	Li et al.^85^	https://github.com/samtools/samtools
SedSeqQuant	This manuscript	https://github.com/jabard89/sedseqquant
DESeq2	Love et al.^86^	https://bioconductor.org/packages/release/bioc/html/DESeq2.html
ViennaRNA	Lorenz et al.^87^	http://rna.tbi.univie.ac.at/
Stellaris^®^ RNA FISH Probe Designer	Biosearch Technologies, Inc.	www.biosearchtech.com/stellarisdesigner
R	Open source	https://cran.r-project.org/mirrors.html
Python	Open source	https://www.python.org/downloads/

## Highlights

- Stresses cause condensation of most mRNAs independent of length
- Newly synthesized mRNAs escape condensation during stress, permitting translation
- mRNP condensates are pervasive and result from translation initiation inhibition
- Ribosome-free mRNA does not cause condensation or stress granule formation
