# Supplementary material for: Transcriptome-wide mRNP condensation precedes stress granule formation and excludes new mRNAs
Source: Mol Cell. Author manuscript; Available in PMC 2025 Dec 2. (PMC12668231; doi:10.1016/j.molcel.2025.11.003)
Supplement: 3 [file NIHMS2121914-supplement-3.pdf]

Supplementary information accompanying "Transcriptome-wide mRNP condensation precedes stress granule formation and excludes new mRNAs"

Glauninger, Bard, Wong Hickernell *et al*. 2025

Methods S1: Modeling and quantification of mRNP condensation, related to Figures 1, S1, and 5, and STAR Methods.

A model of free mRNP sedimentation

Let L be the length of an mRNA under consideration. We seek the functional form of $\mathrm{pSup}(L)$, the proportion in the supernatant after centrifugation, which will depend on many factors. Several of these are experimental and we assume they do not change across samples or mRNAs, such as the spin speed ω, the sample height h, the spin time s, and the viscosity η. Terminal velocity for a particle with mass m and hydration radius r_0 is:

$$v_t(m, r_0) = \frac{m\omega^2 r}{6\pi\eta r_0} = \left(\frac{m}{r_0}\right)\left(\frac{\omega^2 r}{6\pi\eta}\right)$$

We assume that both mass m and hydration radius r_0 scale consistently with length L on average. In the case of mass, we assume proportional scaling: a constant (average) molecular weight per unit length deriving from nucleotides and bound proteins. In the case of hydration radius, our scaling assumption is consistent with standard approaches,[S1] for the radius of gyration R_g scales as L^χ with $\chi = 0.34$ for naked ssRNA due to secondary structure. The hydration radius r_0 will be proportional to R_g assuming a constant density. The scaling relationship will also depend on the geometry of the vessel.

Assuming a constant mass per nucleotide and protein binding per unit length, the mass of an mRNP scales linearly with L. Therefore, the leading term above will tend to scale with L:

$$\frac{m}{r_0} = (c_1 L)^\chi$$

with proportionality constant c_1 taking care of the specific conversion factors (e.g. the molecular weight per nucleotide) and with χ summarizing the scaling.

Then the proportion of the particle species remaining in the supernatant is the proportion of the tube which is more than $v_t s$ from the bottom. That is: $v_t s$ from the bottom. That is:

$$\mathrm{pSup}(m, r_0, h, s) = \begin{cases} 0 & v_t(m, r_0)s \geq h \\ 1 - \frac{v_t(m, r_0)s}{h} & 0 \leq v_t(m, r_0)s < h \end{cases}$$

We can then summarize the dependencies with two constants, as follows.

$$\mathrm{pSup}(L) = 1 - \frac{v_t(m, r_0)s}{h}$$

$$= 1 - \left(\frac{m}{r_0}\right)\left(\frac{\omega^2 r}{6\pi\eta}\right)$$

$$= 1 - c_2(c_1 L)^\chi$$

$$= 1 - \beta L^\chi$$

That is, two parameters should be sufficient to describe the average behavior of free (unclustered) mRNAs of length L. The model fits the average data well; see Figure 1E.

A model of treatment-induced mRNP condensation

To model stress-induced interactions and their effect on pSup, we consider two kinds of interactions, both assumed to be Poisson-distributed with rates which vary across conditions and potentially across transcripts.

The first interaction type is between nucleotides, including direct RNA-RNA interactions and interactions mediated by proteins but dependent on nucleotide content; these are expected to be length-dependent. Consequently, we model them by a per-nucleotide interaction rate ν, such that the probability that an mRNP has k nucleotide-dependent interactions is

$$\Pr(k) = (\nu L)^k e^{-\nu L}/k!.$$

The second interaction type is between molecules and is independent of sequence length, including interactions mediated by the 5’ cap or the 3’ end. We model these as a per-molecule interaction rate μ such that the probability that an mRNP has m such interactions is

$$\Pr(m) = \mu^m e^{-\mu}/m!.$$

These rates permit us to model the probability that an mRNP of a given length L engages in interactions with other mRNPs, which may have varying lengths/sizes and may themselves also engage in additional interactions, making prediction of sedimentation highly complex. To proceed analytically, we make a simplifying assumption: that under the conditions we study, most mRNP condensates are sufficiently large to sediment completely (pSup = 0). Under this assumption, the observed proportion in the supernatant for an mRNA of length L is given by the free-mRNP pSup multiplied by the probability that the mRNP is free. The latter probability is equal to the probability of $n = 0$ per-nucleotide interactions and $m = 0$ per-molecule interactions. Combining the results into a single expression, we have

$$\mathrm{pSup}(L) = (1 - \beta L^{\chi})\, e^{-(\mu + \nu L)}.$$

The mean behavior of transcripts in any particular experiment, in this model, can be derived from only four fitted parameters β, χ, μ, ν and the lengths of all transcripts. Fits of this model are shown in Fig. 1E, where the no-stress sample is modeled with no clustering ($\mu = 0$, $\nu = 0$) and stress samples are modeled with clustering (solid lines) and also in comparison to curves where the per-molecule interaction rate is zero ($\mu = 0$; dashed lines). Fitted values are shown in Figure S1E.

For given values of the two stress-induced-condensation-related parameters μ and ν, the transcript length at which the rate of interactions is equal is given by $L\nu = \mu$ or

$$L_{\mathrm{equiv}} = \frac{\mu}{\nu}.$$

We can then ask what proportion of transcripts are at least this long, $L \geq L_{\mathrm{equiv}}$, such that the stress-induced condensation per-nucleotide (length-dependent) contributes more to condensation than per-molecule (length-independent) interactions. These results are tabulated in Figure S1E.

Models with and without a μ parameter are formally nested, and fits can be compared with an *F* statistic. For both 42°C and 46°C, the model without μ (all interactions are sequence-mediated) can be sharply rejected (*F* = 4134.66, *F* = 21,507.21, $P < 10^{-6}$ in both cases).

Bounds on condensate sizes

How can we put bounds on the average size of condensates, given an observed value of pSup? Any pSup value can be realized in a range of ways, bounded by two extremes. At one extreme, the molecular population is homogenous (all molecules of a particular type are in condensates of the same size), and all clusters have the same probability of remaining in the supernatant, which is pSup. At the other extreme, the molecular population is heterogeneous, with each species of condensate having some proportion in the supernatant $pSup_i$ and proportion of the molecular population q_i. Order the indices *i* such that the lightest species (monomers) has $i=1$

and all condensates are ordered by their size (and hence in the descending order of their pSup) up to *n*. First, if *n*=2, it is clear that the observed $pSup \geq pSup_2$, simply because the average of two numbers is greater than or equal to the smaller of the two numbers. Second, if we define the average of the condensates' pSups as $pSup_{cond}$, we have

$$pSup_{cond} = \frac{1}{1-q_1}\sum_{i>1}^{n} q_i\, pSup_i$$

So that

$$pSup = q_1\, pSup_1 + (1-q_1)pSup_{cond} \geq q_1\, pSup_{cond} + (1-q_1)pSup_{cond} = pSup_{cond}$$

which establishes $pSup \geq pSup_{cond}$ such that in general, pSup is an upper bound on the average condensate size.

Sizes of translation-initiation-inhibited condensates (TIICs)

The observation that TIICs display sizes comparable to polysomes, with a broad distribution centering roughly on a size equivalent to a five-ribosome polysome (Figure 5F) provides useful constraints on the composition of TIICs. A polysome with five ribosomes weighs approximately 17 MDa (each yeast ribosome weighs about 3.3 MDa, and the strong hairpin reporter transcript is ~1.8 kb, which is roughly 0.54 MDa). This size is consistent with TIICs being too small to be visible via standard microscopy, large enough to be detected by sedimentation assays, and too large to be a single mRNP. Given that the strong hairpin transcript is 1800 nucleotides long, to reach 17 MDa would require binding of one 65 kDa RNA-binding protein (e.g., Pab1) every 7 nucleotides, which is unphysical (e.g., Pab1 requires 12 nucleotides for full-affinity binding). Indirect binding of larger complexes is formally possible, but would need to produce multi-megadalton mRNP complexes even on short mRNAs. However, clustering (condensation) of multiple mRNPs is already known to occur in the context of stress granules and P-bodies, and provides a simple explanation for all of these observations.

Supplementary Figures

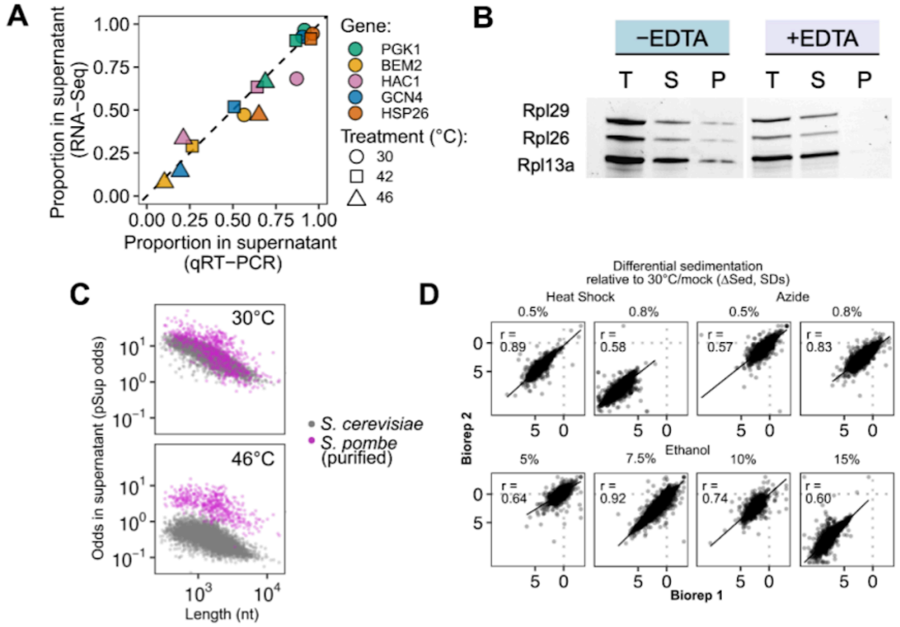

E

$$\mathrm{pSup}(L) = (1-\beta L^{\chi})e^{-\mu}e^{-\nu L}$$

Condition	β	χ	μ	ν	L_{equiv}	Proportion of transcripts with $L \geq L_{\mathrm{equiv}}$
30 °C	2.23×10^{-4}	0.858	–	–	–	–
42 °C	"	"	0.249	9.50×10^{-5}	2617	0.168
46 °C	"	"	1.10	1.79×10^{-4}	6158	0.007
42 °C (ν only)	"	"	0	2.47×10^{-4}	–	–
46 °C (ν only)	"	"	0	7.26×10^{-4}	–	–

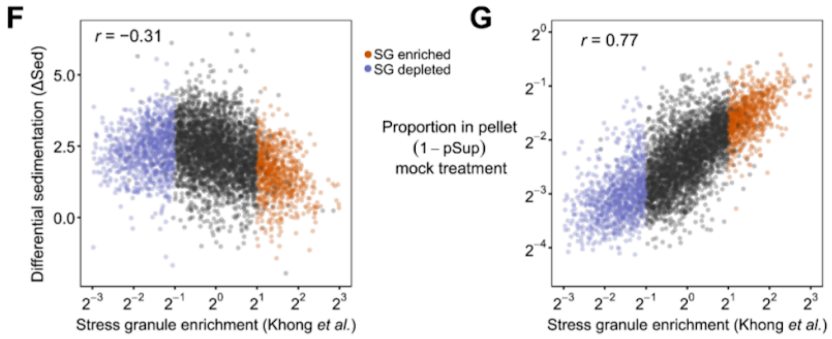

Figure S1: **Sed-seq captures previously unreported mRNA condensation largely driven by length-independent mechanisms, related to Figure 1.** (A) The measurements of mRNA proportion in supernatant (pSup) from RNA sequencing match pSups calculated using spike-in-normalized abundances quantified by RT-qPCR. (B) EDTA prevents sedimentation of polysomes, as shown by western blot against three ribosomal proteins. See also Fig. 5G. Gel cropped for clarity. (C) Purified mRNA (*S. pombe* total RNA) spiked into lysate from unstressed yeast cells recapitulates length-dependent sedimentation of free mRNA, and remains largely free even when spiked into lysate from severely stressed yeast cells. (D) Across stresses, differential sedimentation (ΔSed) of biological replicates correlate well (*r*, Pearson correlation coefficient). (E) Fitted parameters for the four-parameter model shown; see Supplementary Text. (F) The reported enrichment of transcripts in azide-induced stress granules (Khong *et al.* 2017) and our measured differential sedimentation transcript sedimentation during azide stress show mild negative correlation. (G) Most of the variation in stress-granule depletion in Khong *et al.* 2017 can be explained by sedimentation of free mRNPs due to their length, and thus can be closely reproduced using only Sed-seq data from unstressed (mock-treated) cells.

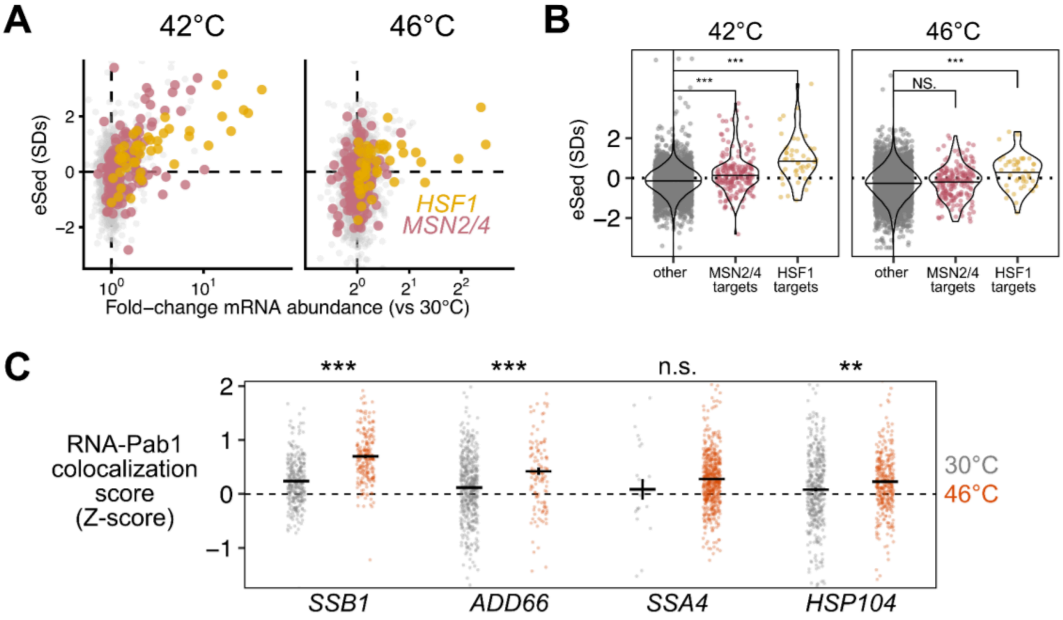

Figure S2: Induced transcripts escape condensation biochemically and microscopically during heat shock, related to Figure 2. (A) Induced Hsf1 targets and Msn2/4 targets relatively escape condensation. (B) Hsf1-regulated transcripts escape condensation at both 42°C and 46°C, while MSN2/4 targets escape at 42°C (violin plot; median, each point represents one transcript species). (Wilcoxon rank sum test: N.S. = P > 0.05, *** = P < 0.001) (C) Colocalization was quantified by comparing the intensity of the Pab1 channel in regions with mRNA foci to the intensity of the Pab1 channel in random regions in each cell. The colocalization score is calculated separately per cell (mean ± SE). (Wilcoxon rank sum test, N.S.: $P \geq 0.05$; **: $P < 0.01$; ***: $P < 0.001$)

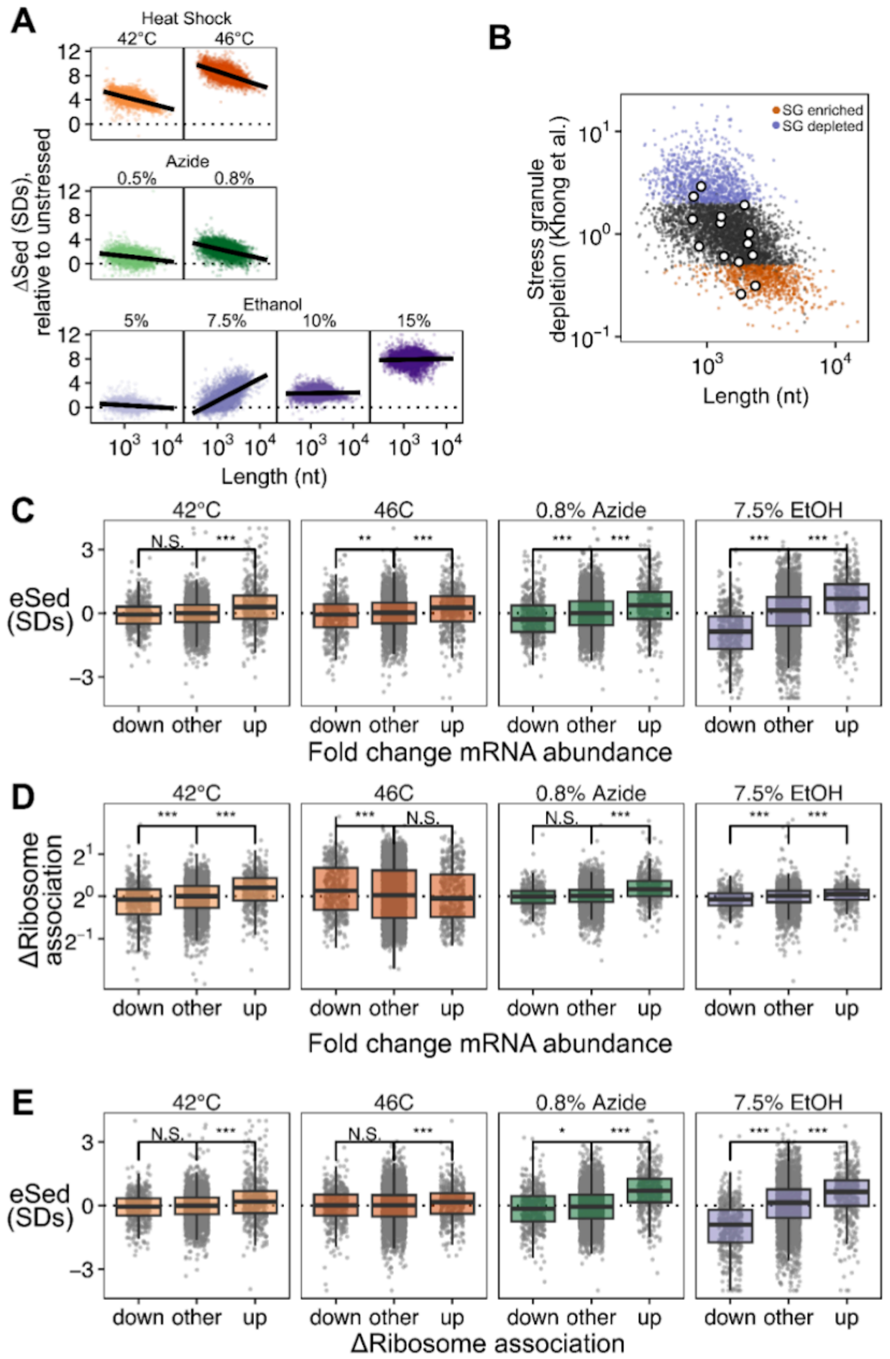

Figure S3: Rather than length, transcriptional and translational induction predict condensation escape, related to Figure 3. (A) Length dependence of the change in sedimentation compared across stresses. In general, shorter transcripts increase in sedimentation more than long ones. (B) Induced transcripts are not particularly depleted from stress granules based on data from Khong *et al.*, 2017. Points in white are the top 1% of induced transcripts during azide stress. (C) Comparison of escape from sedimentation (eSed) grouped by the 10% most induced and repressed transcripts during stress (in C-E: boxplot; median ± quartile). (D) Comparison of the change in ribosome association grouped by the 10% most induced and repressed transcripts during stress. (E) Comparison of eSed grouped by the top and bottom 10% transcripts with changing ribosome association during stress. (Wilcoxon rank sum with Bonferroni correction: N.S. = $P \geq 0.05$, ** = $P < 0.01$, *** = $P < 0.001$)

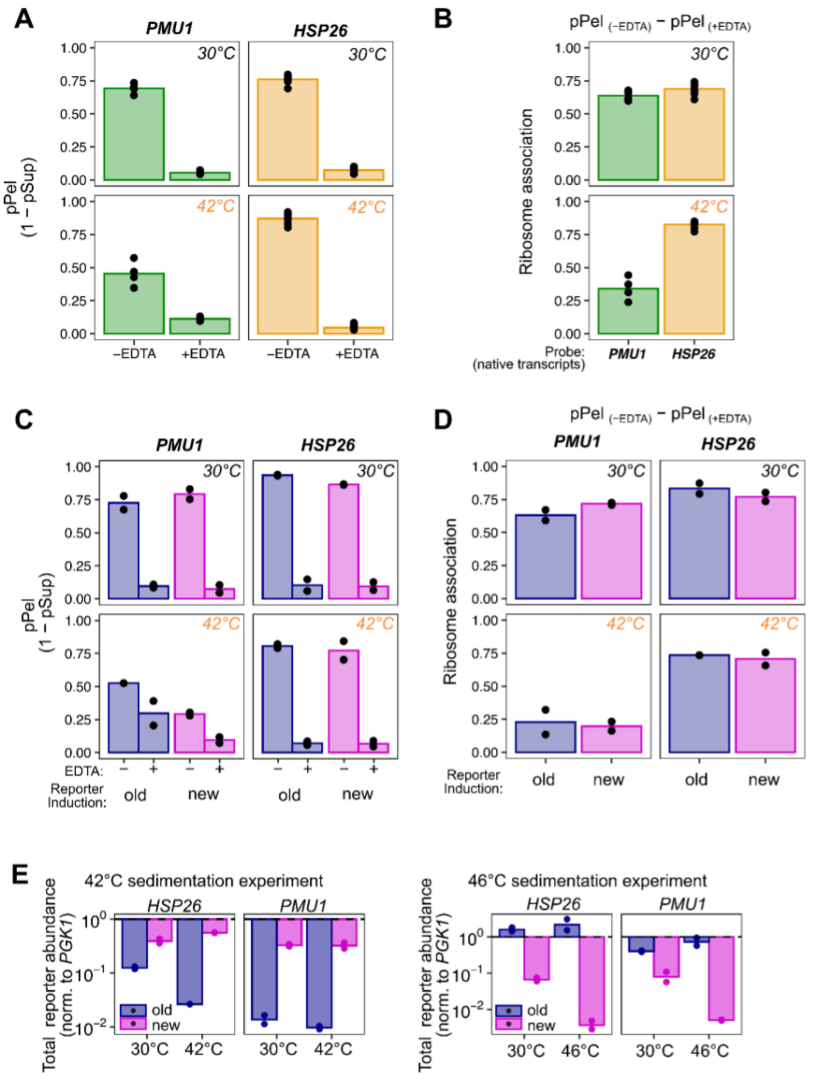

Figure S4: Sedimentation assays to measure condensation and ribosome occupancy of reporter transcripts, related to Figure 4. (A) Cellular lysate from stressed (20 minutes at 42°C) or unstressed yeast was centrifuged through a 1M sucrose cushion with and without EDTA. The proportion of mRNA transcripts in the pellet was quantified with qPCR, normalized to spike-in RNA (in A-D: summarized as mean). (B) In the absence of EDTA, transcripts can pellet either because they are bound to ribosomes or because they are condensed, however ribosomes fall off transcripts in the presence of EDTA. Thus to calculate the ribosome occupancy, the pPel (+EDTA) is subtracted from the pPel (−EDTA). (C,D) The same samples as in (A) and (B), but tracking the abundance of the *HSP26* and *PMU1* reporters induced before stress (old) or after stress (new). (E) Total abundance of the reporter mRNAs, measured by qPCR and normalized to native *PGK1* abundance (summarized as geometric mean).

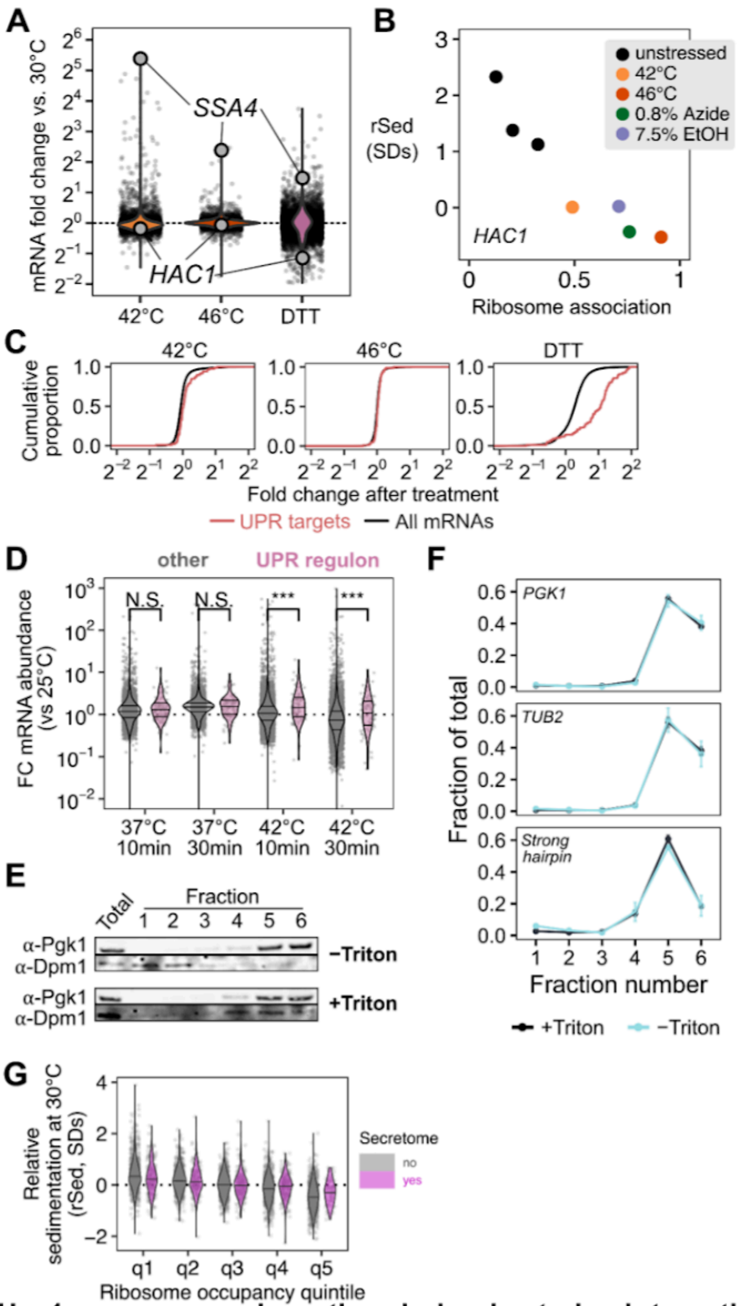

Figure S5: Hac1 escapes condensation during heat shock to activate the UPR, and TIICs are not membrane bound, related to Figure 5. (A) *HAC1* is not induced during temperature or DTT stress (violin plot). (B) *Hac1* mRNA increases in ribosome association and correspondingly decreases in relative sedimentation during stress. (C) UPR targets are slightly upregulated at 42°C and strongly upregulated in response to DTT treatment. (D) Reanalysis of Muhlhofer *et al*. 2020 RNA-seq data confirms that the UPR is upregulated after a 42°C stress but not a 37°C stress (violin plot; median ± quartile). (E) In a membrane pelleting assay, Triton X-100 disrupts the interaction between membranes and normally membrane-bound Dpm1 as measured by western blot across the fractions. Gel cropped for clarity. (F) However, the transcript of the strong hairpin reporter, quantified by qPCR, is not found in the membrane-bound fraction with or without Triton X-100 (mean ± SE). (G) Relative sedimentation of transcripts at 30°C, categorized by ribosome occupancy (q1 is least, q5 is most) and whether the encoded protein is secreted or not (annotations from Costa et al. 2018) (violin plot; median).

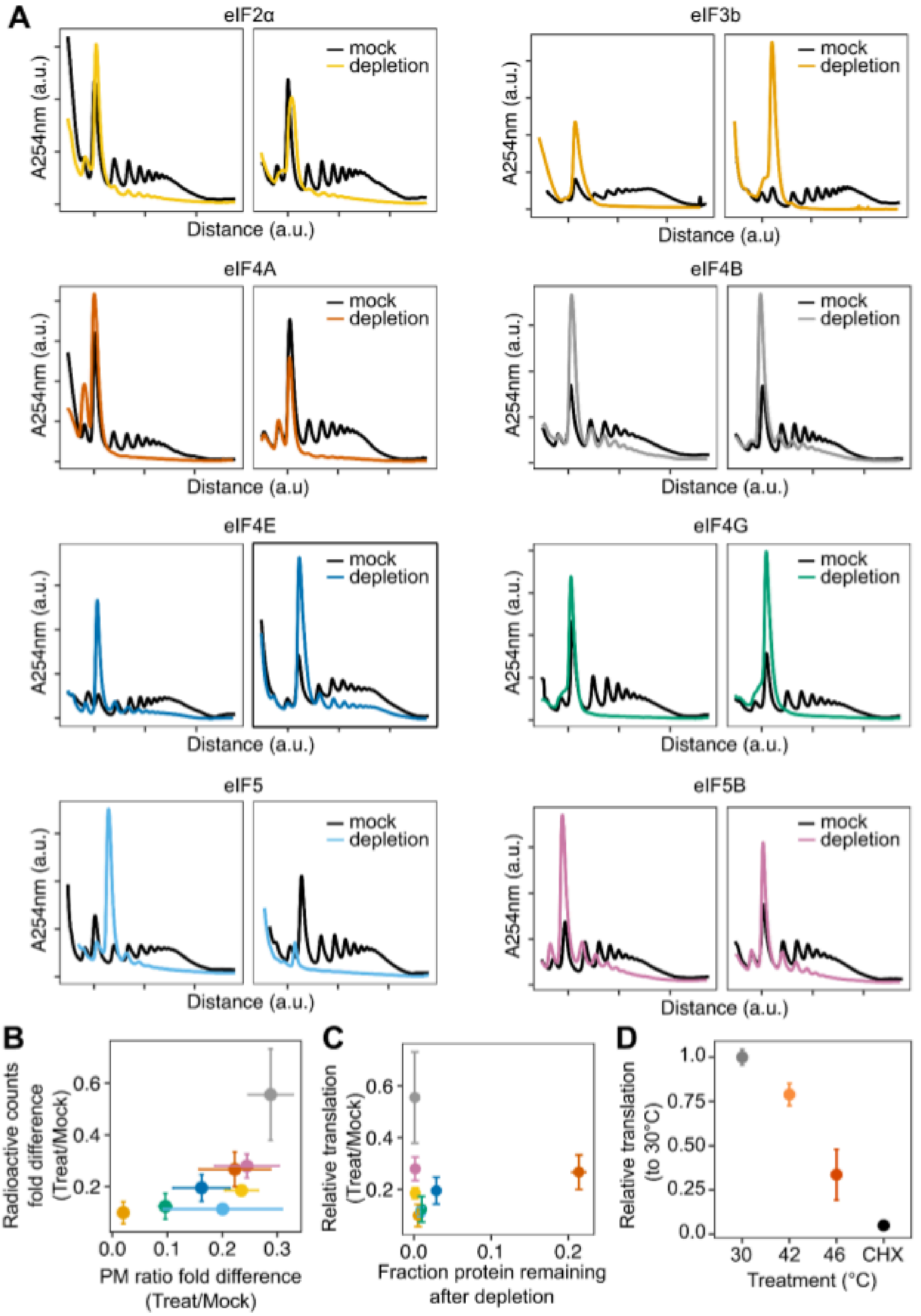

Figure S6: Depleting initiation factors causes global translational shutdown, related to Figure 6. (A) Polysome profiles of AID depletion strains (B) Comparing the different measures of translation, radiolabeled amino acid incorporation and polysome profiling (mean ± SD). (C) Comparing relative translation via radiolabelling vs. the amount of each protein remaining after the AID depletion (mean ± SD). (D) Measures of relative translation via radiolabeled amino acid incorporation after 15 minutes of 30°C, 42°C, 46°C, or 100 μg/mL CHX (mean ± SD).

Table S3: List of RT-qPCR primers and probes, related to STAR Methods:

Gene	Forward primer	Reverse primer	TaqMan probe
PGK1	CTCACTCTTCTATGGTCGGTTTC	AATGGTCTGGTTGGGTTCTC	/56-FAM/TTTCCAACA/ZEN/AGAAACCGGCAGCAC/3IABkFQ/
BEM2	GACTCCCATTGCGTACAAGAA	TCGAGACTGAGGAGGTGATAAA	/56-FAM/TCATTGTTG/ZEN/CCTTCACGTTCCTCGT/3IABkFQ/
HAC1	TGGAAATGACTGATTTTGAACTAACT	CGATCCTTCGCTGTTCCTTT	/56-FAM/TCGCAATCG/ZEN/AACTTGGCTATCCCT/3IABkFQ/
GCN4	ACGCTGTAGTGGAATCTTTCTT	CGTCAGTGGTAACTGGAATGT	/56-FAM/TCGTCAAGC/ZEN/ACTGATTCAACTCCAATGT/3IABkFQ/
HSP26	CTTGTCCCTGTTCCCATCTG	GACACCAGGAACCACGACTT	/56-FAM/CGGTTTCCC/ZEN/TAGAAGTGTCGCAGTT/3IABkFQ/
Nanoluciferase spike in	AGAGGACTTTGTGGGTGATTG	GTTACGGATACGCCAAGGTT	/56-FAM/AGGAGGCGT/ZEN/GTCATCCTTGTTTCA/3IABkFQ/
yo-Nanoluciferase reporter	GAACAAGGTGGTGTTTCTTCTTT	TGACCCATTTGATCACCAGATAA	/56-FAM/ACGTTGAAT/ZEN/AGGAGTAACAGAAACACC/3IABkFQ/
Clover	GGTGATGCAACTAACGGTAAAC	AAGCTACACCATACCCGAAAG	/56-FAM/TTGCCTGTA/ZEN/CCATGGCCAACGTTA/3IABkFQ/

References

[S1] Yoffe, A.M., Prinsen, P., Gopal, A., Knobler, C.M., Gelbart, W.M., and Ben-Shaul, A. (2008). Predicting the sizes of large RNA molecules. Proc. Natl. Acad. Sci. U. S. A. *105*, 16153–16158.
